# Supplementary material for: Gestational route to healthy birth (GaRBH): protocol for an Indian prospective cohort study
Source: BMJ Open. 2019 May 1;9(4):e025395. doi: 10.1136/bmjopen-2018-025395 (PMC6501957; doi:10.1136/bmjopen-2018-025395)
Supplement: Author's manuscript [file bmjopen-2018-025395.draft_revisions.pdf]

BMJ Open

BMJ Open is committed to open peer review. As part of this commitment we make the peer review history of every article we publish publicly available.

When an article is published we post the peer reviewers' comments and the authors' responses online. We also post the versions of the paper that were used during peer review. These are the versions that the peer review comments apply to.

The versions of the paper that follow are the versions that were submitted during the peer review process. They are not the versions of record or the final published versions. They should not be cited or distributed as the published version of this manuscript.

BMJ Open is an open access journal and the full, final, typeset and author-corrected version of record of the manuscript is available on our site with no access controls, subscription charges or pay-per-view fees (http://bmjopen.bmj.com).

If you have any questions on BMJ Open's open peer review process please email info.bmjopen@bmj.com

BMJ Open

Gestational Route to Healthy Birth (GaRBH): a prospective cohort protocol

Journal:	*BMJ Open*
Manuscript ID	bmjopen-2018-025395
Article Type:	Protocol
Date Submitted by the Author:	12-Jul-2018

Complete List of Authors:	Gupta, Vipin; University of Delhi, Department of Anthropology<br>Saxena, Ruchi; Sardar Patel Medical College, Department of Obstetrics and Gynaecology<br>Walia, Gagandeep; Public Health Foundation of India<br>Agarwal, Tripti; Public Health Foundation of India<br>Vats, Harsh; Department of Anthropology, University of Delhi<br>Dunn, Warwick; School of Biosciences, Phenome Centre Birmingham and Institute of Metabolism and Systems Research, University of Birmingham, UK<br>Relton, Caroline; MRC Integrative Epidemiology Unit and Bristol Medical School, University of Bristol, UK<br>Sovio, Ulla; University of Cambridge, Obstetrics and Gyneacology<br>Papageorghiou, Aris; University of Oxford<br>Davey Smith, George; MRC Integrative Epidemiology Unit and Bristol Medical School, University of Bristol, UK<br>Khadgawat, Rajesh; All India Institute of Medical Sciences, New Delhi<br>Sachdeva, Mohinder; Department of Anthropology, University of Delhi

Keywords:	Birth cohort, India, Developmental origin, Foetal growth, Metabolomics in Pregnancy, Epidemiology < TROPICAL MEDICINE

SCHOLARONE™
Manuscripts

Title:

Gestational Route to Healthy Birth (GaRBH): a prospective cohort protocol

Vipin Gupta[1], Ruchi Saxena[2], Gagandeep Kaur Walia[3], Tripti Aggarwal[3], Harsh Vats[1], Warwick Dunn[4], Caroline Relton[5], Ulla Sovio[6], Aris Papageorghiou[7], George Davey Smith[5], Rajesh Khadgawat[8] and M. P. Sachdeva[1]

[1]Department of Anthropology, University of Delhi, India
[2]Sardar Patel Medical College, Bikaner, Rajasthan, India
[3]Public Health Foundation of India, India
[4]School of Biosciences, Phenome Centre Birmingham and Institute of Metabolism and Systems Research, University of Birmingham, UK
[5] MRC Integrative Epidemiology Unit and Bristol Medical School, University of Bristol, UK
[6]Department of Obstetrics and Gynaecology, School of Clinical Medicine, University of Cambridge, UK
[7]Nuffield Department of Women's and Reproductive Health, University of Oxford, UK
[8]Department of Endocrinology, Metabolism and Diabetes, All India Institute of Medical Sciences, India

Corresponding Author

Dr. Vipin Gupta
Department of Anthropology,
University of Delhi, Delhi-110007, India
Email: udaiig@gmail.com
Mobile: 9899119242

For peer review only

Abstract

Introduction: Pregnancy is characterized by a high rate of metabolic shifts from early to late phases of gestation in order to meet the raised physiological and metabolic needs. The change in levels of metabolites is influenced by gestational weight gain (GWG) which is an important characteristic of healthy pregnancy. Inadequate or excessive GWG has short and long term implications on maternal and child health. System-wide exploration of gestational metabolism is required for understanding the quantitative changes in metabolite levels during the course of pregnancy. Therefore, our aim is to study trimester-specific variation in levels of metabolites in relation to GWG and its influence on foetal growth and newborn anthropometric traits at birth.

Methods and analysis: A prospective longitudinal study is planned (start date: February, 2018) on pregnant women that are being recruited in the first trimester (baseline) and followed in subsequent trimesters and at the time of delivery (total 3-follow-ups). The study is being conducted in a hospital located in Bikaner district (66% rural population), Rajasthan, India. The estimated sample size is of 1000 mother-offspring pairs. Information on gynaecological and obstetric history, socioeconomic position, diet, physical activity, tobacco and alcohol consumption, depression, anthropometric measurements, biochemistry and metabolomics is being collected in each trimester using standardized methods. Mixed effects regression models will be used to assess the role of gestational weight in influencing metabolite levels in each trimester. The association of maternal levels of metabolites with foetal growth, offspring's weight and body composition at birth will be investigated using regression modelling.

Ethics and dissemination: The study has been approved by the ethics committees of the Department of Anthropology, University of Delhi and Sardar Patel Medical College, Bikaner, Rajasthan. We are taking written informed consent after discussing the various aspects of the study with the participants in the local language.

Key words: Birth cohort, India, Metabolomics in Pregnancy, Foetal growth, Epidemiology and Developmental origin

Word Count: 5004

For peer review only - http://bmjopen.bmj.com/site/about/guidelines.xhtml

Article Summary

Strength and Limitations

- This is a longitudinal study in India which is examining trimester specific metabolomic changes during the course of pregnancy and their influence on foetal growth.
- This is an Indian study that is collecting objective measurements on pregnant women during each trimester (1st, 2nd and 3rd trimester) and their offspring at the time of birth.
- Newborns will be intensively phenotyped for the levels of metabolites and body composition.
- The majority of study participants are being recruited from a rural area which is in contrast to other Indian cohorts.
- Lack of information on pre-pregnancy measurements will restrict our analysis to pregnancy only.

For peer review only - http://bmjopen.bmj.com/site/about/guidelines.xhtml

For peer review only

Introduction

The profound metabolic changes during healthy pregnancy ensure optimum foetal development and appropriate nutrient allocation between the mother and foetus.[1,2] Such metabolic adjustments are required for meeting raised physiological needs of pregnancy and for provision of additional energy to support labour and lactation.[1] The concept of *"the developmental origins of health and disease"* suggests that exposures during critical periods *in utero* can lead to *"programmed"* structural and/or functional alterations that can transmit to the next generation and predispose them to disease at later age.[3,4] Pregnancy is dominated by metabolic changes to meet the needs of foetal growth and development whilst protecting the health of the mother. For example, a study on 115 women with normal pregnancy has found that higher circulating fatty acids are associated with lower blood pressure and more favourable metabolic phenotype of the mothers.[5] The disparity in metabolic changes during pregnancy may influence foetal growth, and can increase the likelihood of adverse metabolic profiles across the life-course for both mother and the offspring through foetal programming.[6] For instance, increased transport of glucose and free fatty acids to the foetal-placental unit can lead to adverse foetal growth (i.e. macrosomia).[7,8]

The system-wide understanding of metabolism requires us to capture phenotypic changes of the human *metabolome,* which comprises the full spectrum of metabolites present in all cells, tissues or fluids of the human body,[9,10,11] in different physiological states (i.e. puberty, pregnancy, lactation and pre/post-menopause). The metabolome can be considered as an intermediate phenotypic mixture of metabolites encoded by our genome and influenced by specific environmental exposures, and it shapes an individual's *metabolic phenotype or* metabotype.[10,11,12] It can be studied through the holistic approach of *metabolomics* that provides a quantitative fingerprint of all metabolites present in cells, tissues or organism and can be measured using high throughput technologies.[13,14] The field of metabolomics is important for uncovering quantitative metabolic trajectories of normal and abnormal pregnancies. The quantitative estimation of longitudinal metabolomic changes during gestation are globally few in number[15,16,17] and certainly not characterized in the Indian population.

Metabolomic changes in pregnancy

The incomplete understanding of the complex nature of metabolic processes during pregnancy has generated considerable interest in exploring the effect of gestational characteristics on metabolomic changes during pregnancy and its impact on offspring health. Hyperglycemia during pregnancy is one of the widely studied examples of pregnancy induced metabolic stress which increases maternal risk of type 2 diabetes and also predisposes the developing foetus to poor metabolic health.[18] Studies have also assessed the metabolomics of pregnancy complications,

For peer review only - http://bmjopen.bmj.com/site/about/guidelines.xhtml

such as, gestational diabetes,[19,20] placental abruption[21], and birth outcomes, such as preterm birth[22], small for gestational age[23] and large for gestational age.[24]

An exploratory study has investigated the changes in metabolite levels during pregnancy among women with gestational diabetes and found elevated levels of branch chain amino acids, leucine, isoleucine, and valine.[25] The catabolic nature of third trimester is represented by a decrease in maternal glucose levels and substantial rise in the levels of fasting insulin which in turn can lead to insulin resistance.[26] Moreover, circulating levels of lipids and lipoproteins rise 2-3 fold in late pregnancy.[27] Maternal amino acid levels change longitudinally due to placental uptake and foetal transfer across the trimesters.[15] There is an increase in energy production through the TCA cycle during the course of gestation along with concomitant decreases in free carnitine and acetylcarnitine and increase in carnitine palmitoyltransferase-1 activity and β-hydroxybutyrate levels.[15]

Relationships between maternal gestational weight gain and newborn health

Gestational weight gain (GWG) is a complex biological trait which is influenced by maternal physiology, maternal and foetal metabolism and placental metabolism.[28] There are short or long term implications of inadequate or excessive GWG which influence the trade-off between maternal and child health outcomes.[28,29,30,31.32] For example, inadequate weight gain is associated with high risks of low birth weight, small for gestational age, and preterm birth,[33] whereas excessive GWG has been associated with large for gestational age, gestational diabetes, caesarean section and postpartum weight retention.[34] Further, high pre-pregnancy maternal BMI is associated with perturbations to maternal metabolome across gestation.[35]

Maternal GWG is a good indicator of foetal nutrition.[36] Any amount of weight gain in the first 14 weeks and high rate of weight gain (>500g/week) in weeks 14 to 36 are associated with raised adiposity in the offspring.[37] GWG in the first trimester is associated with offspring body mass index from infancy to early childhood whereas excessive GWG (i.e. ≥500g/week) in 2nd and 3rd trimesters is associated with increased birth weight.[38] The excessive GWG in the first half of the pregnancy (<20 weeks) is associated with higher birth weight and excessive body fat in newborns compared to the second half of pregnancy.[39] Further, Gaillard et al.[40] observed that early GWG was associated with increased adiposity levels and adverse cardio-metabolic profile in childhood independent of pre-pregnancy weight. A study that investigated the role of trimester specific GWG on newborn anthropometry has found that women with an excessive rate of GWG in mid/late pregnancy deliver the heaviest babies.[41]

Excessive GWG in early pregnancy, which is actually maternal fat deposition, may lead to maternal dysmetabolism[42,43] and influence the levels of maternal metabolites in subsequent trimesters. A study has found that the levels of triglycerides, HDL-C,

For peer review only - http://bmjopen.bmj.com/site/about/guidelines.xhtml

apoA1 and IL-6 are linearly associated with GWG across all levels of GWG in mid pregnancy.[37] The authors postulated that mothers with high GWG may engage in lifestyles during and after pregnancy that may encourage weight gain.

The continuous variability in the amount of weight gain during pregnancy, with respect to parity, height, geography and ethnicity,[44,45,46] has motivated the research for detecting the predictors of GWG.

Maternal metabolic associations with newborn phenotypes

Maternal pre-pregnancy obesity can predict higher branched chain amino acids and related metabolites in offspring.[47] The metabolic shift from first to third trimester can influence newborn phenotypes. A Mendelian randomization study has suggested causal relationships between genetically elevated maternal pre-pregnancy body mass index (1 standard deviation) and blood glucose levels (7.2mg/dl) with higher birth weight (55g and 114g, respectively) of the offspring.[48] A large study related to the effects of maternal metabolic signatures on foetal growth has identified 10 metabolites predictive of foetal growth, and explained 48-53% of total variation in birth weight.[16] Recently, a pilot study has shown that both maternal gestational age (comparable to that estimated by ultrasound) and risk for preterm delivery can be predicted by measuring cell-free RNA transcripts in maternal blood during pregnancy, highlighting the opportunities offered in the identification of non-invasive blood based biomarkers of pregnancy outcome.[49] The increase in maternal pre-pregnancy BMI is strongly associated with adverse cardio-metabolic profile (very-low-density lipoprotein (VLDL)-lipoproteins, VLDL cholesterol, VLDL-triglycerides, VLDL-diameter, branched/aromatic amino acids, glycoprotein acetyls, and triglycerides) in offspring.[50] It has been shown that maternal hyperglycemia is associated with higher cord blood concentrations of lysophosphatidylcholines in offspring independent of lifestyle intervention among obese women.[51]

Scholtens et al.[52] assessed the impact of maternal hyperglycemia, measured using an oral glucose tolerance test (OGTT), on foetal phenotypes at 28 weeks of gestation. They observed a decrease in amino acids, fatty acids, and products of lipid metabolism whereas a rise in triglycerides and carbohydrates between fasting and 1-hour OGTT samples. Further, the role of maternal glucose, triglycerides and fatty acids in predicting the newborn birth weight and sum of skinfolds thicknesses was also suggested. This confirmed the Freinkel modification of the Pedersen hypothesis i.e. foetal growth and fat accumulation in the setting of maternal hyperglycemia is contributed to by other nutrients in addition to glucose.[53]

Gestational Route to Healthy Birth (GaRBH) cohort

This study aims to quantify differential metabolic variability in relation to maternal weight gain during pregnancy for assessing its influence, as an exposure, on a)

For peer review only - http://bmjopen.bmj.com/site/about/guidelines.xhtml

foetal growth, b) offspring metabolite levels, c) body composition of offspring at birth. The conceptual model is given in Figure-1. We are asking two research questions:

1. Are quantitative changes in levels of metabolites associated with gestational weight gain during pregnancy?

2. Are quantitative metabolic changes during pregnancy associated with foetal growth, offspring levels of metabolites and body composition at birth?

Study objectives

- To detect differential changes in quantitative metabolites between the end of the 1st, 2nd and 3rd trimesters of pregnancy and to examine how these trajectories are associated with maternal weight gain.
- To determine the influence of metabolic changes during pregnancy on foetal growth, body composition (fat mass, lean mass and bone density) and anthropometry at birth (i.e. body length, weight and circumferences).
- To assess the association between maternal metabolic profile during pregnancy and metabolite levels of offspring at birth (in cord blood).

Insert Figure-1

Study area, design and setting

Study Area: This study is being conducted in the government hospital of Sardar Patel Medical College, i.e. "Prince Bijay Singh Memorial Hospital" (PBM), located in the sub-district *Bikaner* which is one of the eight sub-districts ("*Tehsils*") of Bikaner district in Rajasthan state. As per the Census of India,[54] Rajasthan is India's largest state in terms of geographical area, with a population size of 68 million. The population of Bikaner district is 2.3 million (66% is rural) of which 47% are females. In Bikaner, crude birth rate is 26 per 1000 individuals, the total fertility rate is 3 and the infant mortality rate is 60 per 1000 individuals.[55]

Study Design: We have adopted a hospital based longitudinal design for recruiting and following pregnant women until delivery, and their offspring at birth from PBM hospital*, Bikaner.*

Sample size: Studies of fairly large size for detecting associations between longitudinal changes in levels of metabolites in pregnancy and foetal growth, and levels of metabolites of the offspring at birth, are few in number. For example, Ciborowski et al.[56] used 770 samples collected during the first trimester for studying the relationship between maternal metabolic levels with macrosomia and low birth weight. Maitre et al.[57] investigated 34 metabolites on 438 samples recruited during the first trimester and found an association with foetal growth restriction and preterm birth. The majority of studies related to metabolic changes during pregnancy[58,59,60]

For peer review only - http://bmjopen.bmj.com/site/about/guidelines.xhtml

and maternal-foetal medicine[61,62] were based on 30-60 samples.[63,64] Exact power calculations are challenging due to a restricted number of prior studies leading to unknown features.[65] Thus, the proposed sample size must be larger than the majority of informative studies conducted to date and feasible within the cost boundaries for the technology being used.

Tynkkynen et al.[66] studied quantitative urine and serum metabolite data on 995 samples of Northern Finland Birth Cohort for establishing the proof of concept for conducting large scale epidemiological studies in metabolomics using an example of body mass index. A relatively large study (n=800) has found that maternal urine metabolites can independently explain 12% of birth weight after adjusting for all other lifestyle/ clinical factors.[16] They also found an association of trimester specific metabolic changes on foetal weight (change of 1.2% in first trimester and 2.4% in third trimester). Studies have observed that a metabolite level may change from 10 to 30% (even more for some metabolites) from first trimester to third trimester.[15,67]

For a cross-sectional analysis, a sample size of 1000 will be sufficient (with 98% power) to explain 4-5% variation in the level of a metabolite by maternal weight as a predictor, even after correcting for multiple testing (α= 0.0001; for number of metabolites). Moreover, a sample size of 1000 will be sufficient to explain 2% variation in the foetal growth parameters (with 80% power, α= 0.0001) by maternal level of metabolite as a predictor.

Therefore, we propose to recruit 2500 pregnant women during the first trimester in order to measure metabolites on at least 1000 pregnant women (we may recruit >1000 women) in each trimester and their offspring, accounting for loss to follow-up.

Recruitment Strategy

Women visiting the antenatal clinic of the PBM hospital are being screened with the help of a one-page questionnaire to assess their eligibility for the study. We aim to screen around 6000 pregnant women from PBM hospital to reach the targeted sample size. The participants will have to satisfy the eligibility criteria (given below) before being recruited at baseline (Figure-2):

1. Less than or equal to 10 weeks of gestation
2. Age: ≥18 to <35 years
3. Singleton pregnancy
4. Natural conception (i.e. without any fertility treatment)
5. No long-term medication (i.e. not more than two weeks in last one year)
6. No more than one miscarriage in previous pregnancies. In cases where the current pregnancy is the second pregnancy, then the women with miscarriage in their first pregnancy will also be excluded.
7. No previous pregnancy affected by pre-eclampsia/eclampsia
8. No clinical evidence of sexually transmitted disease
9. No hormonal therapy

For peer review only - http://bmjopen.bmj.com/site/about/guidelines.xhtml

The study participants (recruited in first trimester) who develop pregnancy complications in subsequent trimesters that may influence foetal growth (like pre-eclampsia[68], eclampsia, intrauterine growth restriction and gestational diabetes mellitus[69]), will not be excluded, as that would lead to a reduction in the natural variation in the study population. Further, we will not exclude preterm delivery cases due to its relationship with foetal growth[70] and its high burden (i.e. 12.8 million in 2010) in India[71], as it could lead to bias by restricting the sampling to healthy pregnancies which may not represent the general population.

Insert Figure-2

Assessment procedures and instrumentation

Foremost, the study is described by the field investigator to the pregnant women and an informed consent is being taken from the study participants.

Questionnaires

A comprehensive questionnaire has been developed for collecting information on demographic, anthropological (caste, religion and consanguinity), medical disease history, tobacco and alcohol intake, and passive smoking. The validated food frequency questionnaire of "*Indian Migration Study"* is being used for evaluating dietary intake.[72] Maternal physical activity during pregnancy is being assessed using the international physical activity questionnaire (i.e. 7days long).[73, 74,75] The assets based tool of National Family Health Survey-4 is being used to evaluate socioeconomic position.[76] For assessing depression, we are using the Patient Health Questionnaire[77] and Edinburgh Postnatal Depression scale.[78] The WHO-5 questionnaire is being used for collecting information on quality of life.[79] The capability wellbeing, i.e. individual's freedom to function in five key areas of their life: ''stability'', ''attachment'', ''achievement'', ''autonomy'' and ''enjoyment'', is being assessed by using the ICEpop CAPability measure for Adults (ICECAP-A).[80,81,82] Information on maternal environment, gynaecological and obstetrics history is also being collected using tools adapted from INTERGROWTH study.[83,84] Additionally, a structured questionnaire on social factors like relationships with family members, husband position in household, social support during pregnancy, and travel is being used for assessing the quality of social life and adequacy of social support (Table-1).

Insert Table-1

Adult Anthropometry

The assessment of anthropometric traits (weight, height, sitting height, circumferences and skinfolds) are being performed on study participants using standard procedures.[85]

For peer review only - http://bmjopen.bmj.com/site/about/guidelines.xhtml

Weight is being measured when the participant standing straight on the centre of the weighing machine (Tanita, model-HD380), arms hanging freely with minimum clothes and without accessories. Height is being measured as the vertical distance between vertex (highest point on head) and floor when the participant standing straight with feet together on the level floor without footwear, arms resting by the side, with head in eye-ear plane (Frankfort horizontal plane), and heels, buttocks and shoulders projected on the vertical plane using a wall mounted stadiometer (ADE, Germany, model-MZ10023-3). Sitting height is being measured as the maximum distance from the vertex (top of the head) to the base of a flat sitting surface, with legs hanging freely and head in eye-ear plane.

Circumferences are being measured at the specific landmarks when the plane of the tape (ADE, Germany, model-MZ10021) is perpendicular to the long axis of the body and parallel to the floor without tape being pulled too loose or too tight. Waist circumference is being measured at the narrowest point (which is an approximate midpoint) around the waist between the lower rib margin of the last palpable rib and the top of the iliac crest with the help of a measuring tape. Mid-upper arm circumference is being measured as the maximum circumference of the upper arm taken horizontally where the biceps are most developed/widest (at the middle of the biceps). Hip circumference is being measured at the widest part of the hip (buttock) at the level of the trochanterion which is the most superior point on the greater trochanter of the femur, not the most lateral point. Calf circumference is being measured on the right leg as the maximum circumference in a plane perpendicular to the long axis of the calf until the greatest circumference is located when foot is placed on raised platform.

Skinfold measurements are being taken in relaxing muscles by grasping a distinct fold that separates from underlying muscles with the help of Harpenden Skinfold calliper when the jaws of the calliper are perpendicular to the length of the fold on the site of measurement. Skinfold measurements at biceps, triceps, sub-scapular, supra-iliac, medial calf and anterior thigh are being taken to the nearest 0.1mm.

Three readings are being taken for all the anthropometric measures with minimum acceptable difference of 100g, 5mm, 5mm and 2mm between the readings for weight, height, circumferences and skinfolds, respectively. The readings are being recorded to the last completed grams or millimetre.

Infant anthropometry

We will use the protocol of the International Fetal and Newborn Growth Consortium[86] for measuring the infants. Birth weight will be taken within 30 minutes of delivery, without clothes and in stable condition when the infant is placed an electronic weighing scale (ADE, model-M114600, Germany) to the nearest 5g. Infant length will be measured within 12 hours of birth using the Baby length measuring board (ADE range 100–1000 mm, Germany), which has a fixed headboard and moveable

For peer review only - http://bmjopen.bmj.com/site/about/guidelines.xhtml

footboard, placed on a raised flat platform on the level surface. The head of the infant will be positioned in eye-ear plane on the board and holding the infant's legs with the left hand and moving the footboard with the right hand. Head circumference will be measured when the infant is on the lap of the field investigator, with stable head, using a measuring tape (SECA, 212) anchored just above the eyebrows on the forehead, and at the back the tape being positioned over the fullest protuberance of the skull. Three readings will be taken for all the newborn anthropometric traits with minimum acceptable difference of 10g, 5mm, and 2mm between the readings for weight, infant length and circumferences, respectively. The readings will be recorded to the last completed millimetre.

All the equipment used for the assessment of adult and infant anthropometric traits are being calibrated thrice a week with the help of standard weights and measures.

Blood Pressure

Maternal blood pressure is being measured in the sitting position during each trimester after a rest of 5 minutes before taking the first reading, using an Omron blood pressure monitor (model: HBP-1300) and cuff of appropriate size. Three readings will be taken with 2 minutes interval between the readings with maximum acceptable difference of 5mmHg between two readings. The equipment used is being calibrated thrice a week with the readings of sphygmomanometer.

Laboratory tests

The serum samples will be transferred to the Department of Anthropology, University of Delhi, on dry ice for the generation of data on biochemical assays using fasting samples on all the mothers during each trimester and also on their offspring using cord blood. The available auto-analyzer (XL-640, ERBA Mannheim, Czech Republic) in the Department of Anthropology, University of Delhi, India, will be used for biochemistry data generation (i.e. glucose, insulin, lipids, kidney function test (KFT) and liver function tests (LFT)) after ensuring daily calibration. Insulin levels will be estimated using ELISA kits. Further, a reagent lot calibration will also be performed whenever there is a change in lot number of the reagents to estimate the variation in assays between two reagent lots. For internal quality control, one blank per 100 samples and one control sample from *UK National External Quality Assessment Service* (UKNEQAS) of known concentrations will be run in every 200 samples. Moreover, one duplicate and one sample of known concentration from the previous batch will be run per 100 samples for assessing intra and inter assay variation, respectively, to help with analyses in confirming repeatability and reproducibility of biochemistry data. Finally, 5% of the samples will be run in a different laboratory for all the biochemical assays as a measure of external quality control. Moreover, information on participant's blood group, HbS and HIV infection, which are the part of standard protocol of the hospital's antenatal care, will be taken from PBM Hospital.

For peer review only - http://bmjopen.bmj.com/site/about/guidelines.xhtml

Quantitative Metabolomics

The quantitative data on the level of metabolites will be generated through a targeted approach for screening the predefined and known metabolites using a commercial assay i.e. AbsoluteIDQ p400 HR kit [Biocrates Life Sciences AG, Innsbruck, Austria]. It provides data on 400 endogenous metabolites from 11 metabolite classes (i.e. amino acids, biogenic amines, monosaccharides, acylcarnitines, diglycerides, triglycerides, lysophosphatidylcholines, phosphatidylcholines, sphingomyelins, ceramides, and cholesteryl esters). The choice of AbsoluteIDQ p400 HR kit based on proven utility of its previous version of kit (AbsoluteIDQ p180) in several metabolomics based epidemiological studies.[9,87,88,89,90] Our collaborator, the Phenome Centre Birmingham, University of Birmingham, UK, has experience of using this kit in an inter-laboratory trial. For generating data on targeted metabolites a Triple Quadrupole Mass Spectrometry based technology will be used which characterizes a metabolite by its molecular mass, its specific fractionation pattern (tandem mass spectrometry), and high sensitivity.[91] The complete analytical process will be performed using the MetIQ software package, which is an integral part of these kits. The quality control procedure used by Suhre et al.[92] will be followed for generation and cleaning of metabolomic data. One reference sample will be measured three times on each plate across the samples. The quality of metabolite data will be controlled by estimating their coefficient of variation and missing value rates.[15] Outlying samples and data points will be excluded and finally all missing values will be imputed.[93]

Ultrasound Measures

For ultrasound-based measurements relating to foetal growth, we are using the existing standard operating procedures described by INTERGROWTH study[94] to ensure reliable data collection. The data is being collected by trained sonographers of PBM hospital, Bikaner, who are highly familiar with the equipment (ALOKA Prosound Alpha-6, Hitachi Aloka Medical Systems, Tokyo, Japan) and experienced in conducting ultrasound examinations. The intra-observer variation will be assessed by repeat sonographic measurements on 10 participants on the same day. Comparisons will also be made between the readings of two sonographers of the hospital on 10 participants to assess inter-observer variability. The inter-equipment variation in ultrasound measures will be assessed by comparing the PBM results of 10 participants with the radiology laboratory outside the hospital. We will measure the crown-rump length (CRL), biparietal diameter (BPD), occipito-frontal diameter (OFD), head circumference (HC), femur length (FL), anterior-posterior abdominal diameter (APAD). Gestational age based on CRL and foetal weight will be estimated with the help of INTERGROWTH equations.[94,95]

For peer review only - http://bmjopen.bmj.com/site/about/guidelines.xhtml

Estimation of Gestational Age

The accurate estimation of individual level gestational age (GA) during pregnancy is vital for interpreting fetal anatomy and growth patterns and predicting the date of delivery. GA is being calculated from the first day of the last menstrual period (LMP). Moreover, for women providing clear information on LMP (in completed weeks and days) their gestational age is being confirmed with an ultrasound based measurement of crown–rump length (CRL) in the first trimester, using an internationally recognized chart.[94] If the difference between the CRL and LMP estimates is longer than ≤7 days, LMP is being considered valid and taken as the true biological date. In women with a LMP and CRL difference of >7 days, CRL is being considered valid for estimating the gestational age.[96]

Biological sample collection and storage

A 15ml of blood sample is being collected during each trimester from the study participants after overnight fasting (10 hours). We are using EDTA coated, sodium fluoride coated and plain tubes for blood collection for the separation of EDTA-plasma, fluoride-plasma and serum samples, respectively. Serum will be used for determining the levels of metabolites in addition to triglycerides, cholesterol, lipids, KFT, LFT and insulin whereas fluoride-plasma will be used for glucose levels. The layer of WBC after separating plasma will be stored in an EDTA tube for future extraction of DNA for genetic studies. Multiple aliquots of serum and plasma collected at each trimester are being stored in a -80 degree Celsius freezer (Table-2).

We will collect cord blood and placenta at the time of delivery (Table-2) and processed with the help of the standard operating procedure used in the *"Avon Longitudinal Study of Parents and Children"* (ALSPAC), University of Bristol, UK. The cord blood will be collected in EDTA coated, heparin coated and plain tubes (10ml each) and these will be processed for the separation of plasma and serum. The remaining sample in EDTA tube with buffy coat will be stored in a freezer at -80 degree Celsius. In order to stabilize the gene expression, RNA*later* will be added in Heparin tube. Placenta will be processed immediately after delivery and aliquots of tissue sample from maternal and foetal sides, cord tissue, and membrane will be stored in a freezers at -80 degree Celsius (Table-2). All the stored aliquots will be appropriately bar-coded, stored independently in a separate storage box.

Insert Table-2

Body composition

The body composition of infants will be measured using a DXA machine (Osteo Pro Max, BM Technology, Korea) available in PBM Hospital, Bikaner. The machine will be calibrated using infant phantoms at regular intervals. We have developed a

For peer review only - http://bmjopen.bmj.com/site/about/guidelines.xhtml

standard operating procedure with the help of published literature[97,98,99] for neonatal scans of femur, spine and whole body. Newborns will be measured within 48 hours of delivery after feeding in a calm or sleeping state. Infants will be swaddled in a thin cotton blanket in the required position and measurement will be taken without any metals, buttons or pins on the infant. The region of interest (ROI) for anterior-posterior spine will be five vertebral bodies of the lumber region; for femur scan this will include the femoral neck, trochanters, intertrochanteric, and total hip regions. For the whole body scan, the infant will be positioned at the centre of the table, aligned in a straight line from the nose, centre of the body, and down through the knees and toes. Six ROIs will be taken for the whole body scan (head, entire right and left arms, right and left leg regions and trunk).

Data capture, protection and management

The data is being recorded in paper based questionnaires that will be initially entered in Mircosoft Excel, and later transferred to a SQL based database to be developed in collaboration with the University of Bristol, UK. The master data will be stored at multiple locations (at least two) and managed in computers available in the Department of Anthropology, University of Delhi. One unique identification number (UID) is being assigned to each study participant (mothers) that will be common for her questionnaire-based information, biological measurements, blood and other biological samples. Similarly, newborns will also be assigned a UID which is linked to the UID of mothers. The computers will be password protected, and UID linked with personal identifiers necessary for contact and future follow up, will be encrypted and held separately in a different system and backed up regularly. All the biological samples will be collected in pre-assigned bar-coded tubes and vials and will be stored at -80 degree Celsius in Bikaner, Rajasthan. A comprehensive data dictionary will be prepared for all the study variables and the entire data will be regularly checked for outliers, distribution of variables and missingness.

Data analyses

The evaluation of cross-sectional associations of maternal body weight with quantitative metabolite levels in each trimester, independently, will be done using regression-based modelling. Both level of metabolites and gestational weight in each trimester will act as exposure as well as outcome. Variables like socio-economic position, gestational age, parity, diet, smoking, and physical activity will be considered as confounders. Maternal characteristics will be considered as exposures (like trimester specific weight, metabolite levels) to evaluate their association with offspring related outcomes (foetal growth, birth weight, body compositions and metabolite levels). The potential confounders for maternal-offspring associations will be socio-economic position, gestational age, parity, diet, sex of the offspring, smoking and physical activity.

For peer review only - http://bmjopen.bmj.com/site/about/guidelines.xhtml

Mixed effects regression models for repeated measures will be applied for examining longitudinal associations of weight gain from first to third trimester[100] with maternal levels of metabolites during pregnancy and foetal growth.[101] Mediation analyses will be conducted for evaluating the role of maternal metabolites as intermediates between gestational weight gain and offspring's body composition.[102] False discovery rate based multiple testing correction for number of metabolites will be applied.[103] Mediation analysis will be conducted to examine whether the effect of GWG on foetal growth is mediated through alteration in levels of circulating metabolites.

Public health implications

In India, higher prevalence of both malnutrition and over-nutrition,[104,105] increased rates of gestational diabetes[106] and pre-eclampsia[107] are common challenges related to maternal-foetal health. Moreover, India has high rates of birth defects, i.e. over 60 per 1000 live births[108], higher number of infants born small for gestational age,[71] still births,[109] and more than 45% of children under 5 years of age are stunted[110] which need to be addressed through translational research. The metabolomic approach will enable the identification of biochemical markers predictive for undesirable birth outcomes.

The role of the intrauterine environment, through foetal overnutrition, has been proposed in considering determination of higher birth weight, childhood obesity, impaired glucose tolerance, and dyslipidemia[111,112,113]. The knowledge of trimester-specific quantitative levels of metabolites will uncover mechanistic pathways relevant for optimum foetal growth and healthy birth outcomes. Understanding of changes in gestational metabolism will provide many opportunities for detecting potentially relevant metabolites for planning effective interventions during pregnancy.

Ethics and dissemination

The study has been approved by the ethics committees of the Department of Anthropology, University of Delhi and Sardar Patel Medical College, Bikaner, Rajasthan. We are taking written informed consent after discussing the various aspects of the study with the participants in the local language. The findings of the study will be disseminated through peer reviewed national and international journals and through conferences and seminars.

Acknowledgements

We are thankful to the study participants and to the administration of PBM hospital, Bikaner, Rajasthan for facilitating the study.

Authors' Contributions

VG conceived the study concept with the help of GDS. VG developed the funding proposal, applied for funding and initiated the writing of the protocol paper. VG, GKW

For peer review only - http://bmjopen.bmj.com/site/about/guidelines.xhtml

and MPS developed the study tools and procedures. VG, RS, RK, MPS and GKW coordinated the implementation and running of the study. GDS has advised in developing the study protocols and quality control and assurances. WD has provided expert opinion on metabolomic data generation and quality control. AP has provided expert opinion on ultrasound measures. GDS, CL, US, WD and VG have contributed to the statistical analysis plan and data interpretation. VG, RS, TA and HV contributed to data collection and field management. All authors contributed in the revision and approval of the final version of the manuscript.

Funder's statement: This study has been supported by Wellcome/DBT India Alliance, Hyderabad, India [IA/CPHI/16/1/502623].

Competing interest statement: None

For peer review only

Table-1:Measures, tools and time points of data collection

	Mother			Delivery
	1st Trimester	2nd Trimester	3rd Trimester	Offspring
**Gynaecological and Obstetric**	✔	✔	✔	-
**Socio-economic position**	✔	✔	✔	-
**Anthropological (caste and religion)**	✔	✔	✔	-
**Education**	✔	✔	✔	-
**Marital status**	✔	✔	✔	-
**Smoking**	✔	✔	✔	-
**Alcohol drinking**	✔	✔	✔	-
**Medication**	✔	✔	✔	-
**Disease history**	✔	✔	✔	-
**Maternal Environment**	✔	✔	✔	-
**Physical activity (IPAQ)**	✔	✔	✔	-
**Time-Activity pattern**	✔	✔	✔	-
**Food frequency**	✔	✔	✔	-
**Dietary recall (3 days)**	✔	✔	✔	-
**PHQ-12**	✔	✔	✔	-
**Wellbeing (WHO-5)**	✔	✔	✔	-
**Depression (EPNDS)**	✔	✔	✔	-
**Social factors**	✔	✔	✔	-
**Capability wellbeing**	✔	✔	✔	-
**Anthropometry**				
Height/Body length	✔	✔	✔	✔
Sitting height	✔	✔	✔	-
Weight	✔	✔	✔	✔
Circumferences	✔	✔	✔	✔
Skin-folds	✔	✔	✔	✔
**Blood Pressure**	✔	✔	✔	-
**Laboratory tests:** Glucose, Insulin, Hb, Lipids, LFT and KFT, and blood group	✔	✔	✔	✔
**Metabolomic data**	✔	✔	✔	✔
**Ultrasound measures:** CRL, BPD, OFD, HC, FL, AC and APAD	✔	✔	✔	-
**Placenta and cord measures and images**	-	-	-	✔
**Body Composition (DXA Scan)**	-	-	-	✔

IPAQ: International Physical Activity Questionnaire; PHQ: Psychological health Questionnaire; WHO: World Health Organization; EPNDS: Edinburg Post Natal Depression Scale; LFT: liver function tests; KFT: kidney function tests; CRL: Crown-rump length; BPD: Biparietal diameter; OFD: Occipito-frontal diameter; HC: Head Circumference; FL: Femur length; APAD: Anterior-posterior abdominal diameter

For peer review only - http://bmjopen.bmj.com/site/about/guidelines.xhtml

Table-2: Type and number of aliquots of biological samples

	Pregnancy			
	**1st Trimester**	**2nd Trimester**	**3rd Trimester**	**Offspring**
**Blood Samples**				
Serum	**6 aliquots**	**6 aliquots**	**6 aliquots**	**-**
EDTA Plasma	**2 aliquots**	**2 aliquots**	**2 aliquots**	**-**
Fluoride Plasma	**2 aliquots**	**2 aliquots**	**2 aliquots**	**-**
Buffy coat	**1 aliquot**	**1 aliquot**	**1 aliquot**	**-**
**Cord Blood Samples**				
Serum	-	-	-	**4 aliquots**
EDTA Plasma	-	-	-	**3 aliquots**
Heparin Plasma	-	-	-	**3 aliquots**
Buffy coat with RNA*later*	-	-	-	**1 aliquot**
Buffy coat	-	-	-	**1 aliquot**
**Placenta Samples**				
Maternal side tissue	-	-	-	**8 aliquots**
Maternal side tissue with RNA*later*	-	-	-	**4 aliquots**
Foetal side tissue	-	-	-	**1 aliquot**
Foetal side tissue with RNA*later*	-	-	-	**1 aliquot**
Membrane tissue	-	-	-	**1 aliquot**
Cord tissue	-	-	-	**1 aliquot**
Cord tissue with RNA*later*	-	-	-	**1 aliquot**

For peer review only

For peer review only - http://bmjopen.bmj.com/site/about/guidelines.xhtml

References

1. Picciano MF. Pregnancy and lactation: physiological adjustments, nutritional requirements and the role of dietary supplements. *J Nutr* 2003;133:1997S-2002S.
2. Lowe WL, Karban J. Genetics, genomics and metabolomics: new insights into maternal metabolism during pregnancy. *Diabet Med* 2014;31:254-62.
3. Barker DJP. In utero programming of chronic disease. *Clin Sci (Lond)* 1998;95:115-28.
4. Gluckman PD, Hanson MA. Living with the past: evolution, development, and patterns of disease. *Science* 2004;305:1733–6.
5. Carreras-Badosa G, Prats-Puig A, Puig T, et al. Circulating Fatty Acid Synthase in pregnant women: Relationship to blood pressure, maternal metabolism and newborn parameters. *Sci Rep* 2016;6:24167.
6. Hales CN, Barker DJ. The thrifty phenotype hypothesis. *Br Med Bull* 2001;60:5-20.
7. Van Assche FA, Holemans K, Aerts L. Long-term consequences for offspring of diabetes during pregnancy. *Br Med Bull* 2001;60:173-82.
8. Lawlor DA, Lichtenstein P, Fraser A, et al. Does maternal weight gain in pregnancy have long-term effects on offspring adiposity? A sibling study in a prospective cohort of 146,894 men from 136,050 families. *Am J Clin Nutr* 2011;94:142-8.
9. Gieger C, Geistlinger L, Altmaier E, et al. Genetics meets metabolomics: a genomewide association study of metabolite profiles in human serum. *PLoS Genet* 2008;4:e1000282.
10. Suhre K, Gieger C. Genetic variation in metabolic phenotypes: study designs and applications. *Nat Rev Genet* 2012;13:759-69.
11. Wishart DS, Jewison T, Guo AC, et al. HMDB 3.0--The Human Metabolome Database in 2013. *Nucleic Acids Res* 2013;41:D801-7.
12. Nicholson G, Rantalainen M, Li JV, et al. A genome-wide metabolic QTL analysis in Europeans implicates two loci shaped by recent positive selection. *PLoS Genet* 2011;7:e1002270.
13. Fiehn O. Metabolomics--the link between genotypes and phenotypes. *Plant Mol Biol* 2002;48:155-71.
14. Psychogios N, Hau DD, Peng J, et al. The human serum metabolome. *PLoS One* 2011;6:e16957.
15. Lindsay KL, Hellmuth C, Uhl O, et al. Longitudinal Metabolomic Profiling of Amino Acids and Lipids across Healthy Pregnancy. *PLoS One* 2015;10:e0145794.
16. Maitre L, Villanueva CM, Lewis MR, et al. Maternal urinary metabolic signatures of fetal growth and associated clinical and environmental factors in the INMA study. *BMC Med* 2016;14:177.
17. Gong S, Sovio U, Aye ILMH, et al. Placental polyamine metabolism differs by fetal sex, fetal growth restriction and preeclampsia. JCI Insight (in press).

For peer review only - http://bmjopen.bmj.com/site/about/guidelines.xhtml

18. Ruchat SM, Hivert MF, Bouchard L. Epigenetic programming of obesity and diabetes by in utero exposure to gestational diabetes mellitus. *Nutr Rev* 2013;71:S88-94.
19. Gao J, Xu B, Zhang X, et al. Association between serum bile acid profiles and gestational diabetes mellitus: A targeted metabolomics study. *Clin Chim Acta* 2016;459:63-72.
20. Lu L, Koulman A, Petry CJ, et al. An Unbiased Lipidomics Approach Identifies Early Second Trimester Lipids Predictive of Maternal Glycemic Traits and Gestational Diabetes Mellitus. *Diabetes Care* 2016;39:2232-2239.
21. Gelaye B, Sumner SJ, McRitchie S, et al. Maternal Early Pregnancy Serum Metabolomics Profile and Abnormal Vaginal Bleeding as Predictors of Placental Abruption: A Prospective Study. *PLoS One* 2016;11:e0156755.
22. Li J, Lu YP, Reichetzeder C, et al. Maternal PCaaC38:6 is Associated With Preterm Birth - a Risk Factor for Early and Late Adverse Outcome of the Offspring. *Kidney Blood Press Res* 2016;41:250-7.
23. Powell KL, Carrozzi A, Stephens AS, et al. Utility of metabolic profiling of serum in the diagnosis of pregnancy complications. *Placenta* 2018;66:65-73.
24. Dessì A, Marincola FC, Pattumelli MG, et al. Investigation of the ^{1}H-NMR based urine metabolomic profiles of IUGR, LGA and AGA newborns on the first day of life. *J Matern Fetal Neonatal Med* 2014;27:13-9.
25. Chorell E, Hall UA, Gustavsson C, Berntorp K, Puhkala J, Luoto R, Olsson T, Holmäng A. **Pregnancy** to postpartum transition of serum metabolites in women with gestational diabetes. Metabolism. 2017 Jul;72:27-36.
26. Lain KY, Catalano PM. Metabolic changes in pregnancy. *Clin Obstet Gynecol* 2007;50:938–48.
27. Diareme M, Karkalousos P, Theodoropoulos G, et al. Lipid profile of healthy women during normal pregnancy. *Journal of Medical Biochemistry* 2009;28:152–60.
28. IOM (Institute of Medicine)/NRC (National Research Council). Weight gain during pregnancy: reexamining the guidelines. In: Rasmussen KM, Yaktine AL, eds. *Food and Nutrition Board and Board on Children, Youth, and Families*. National Academies Press (US), 2009.
29. Fraser A, Tilling K, Macdonald-Wallis C, et al. Associations of gestational weight gain with maternal body mass index, waist circumference, and blood pressure measured 16y after pregnancy: the Avon Longitudinal Study of Parents and Children (ALSPAC). *Am J Clin Nutr* 2011;93:1285-92.
30. Poston L. Gestational weight gain: influences on the long-term health of the child. *Curr Opin Clin Nutr Metab Care* 2012;15:252-7.
31. Drehmer M, Duncan BB, Kac G, et al. Association of second and third trimester weight gain in pregnancy with maternal and fetal outcomes. *PLoS One* 2013;8:e54704.
32. Goldstein RF, Abell SK, Ranasinha S, et al. Association of Gestational Weight Gain With Maternal and Infant Outcomes: A Systematic Review and Meta-analysis. *JAMA* 2017;317:2207-2225.

For peer review only - http://bmjopen.bmj.com/site/about/guidelines.xhtml

33. Mannan M, Doi SA, Mamun AA. Association between weight gain during pregnancy and postpartum weight retention and obesity: a bias-adjusted meta-analysis. *Nutr Rev* 2013;71:343–52.
34. Oken E, Kleinman KP, Belfort MB, et al. Associations of gestational weight gain with short- and longer-term maternal and child health outcomes. *Am J Epidemiol* 2009;170:173-80.
35. Hellmuth C, Lindsay KL, Uhl O, et al. Association of maternal prepregnancy BMI with metabolomic profile across gestation. *Int J Obes (Lond)* 2017;41:159-169.
36. Lawlor DA. The Society for Social Medicine John Pemberton Lecture 2011. Developmental overnutritiondan old hypothesis with new importance? *Int J Epidemiol* 2013;42:7e29.
37. Fraser A, Tilling K, Macdonald-Wallis C, et al. Association of maternal weight gain in pregnancy with offspring obesity and metabolic and vascular traits in childhood. *Circulation* 2010;121:2557-64.
38. Karachaliou M, Georgiou V, Roumeliotaki T, et al. Association of trimester-specific gestational weight gain with fetal growth, offspring obesity, and cardiometabolic traits in early childhood. *Am J Obstet Gynecol* 2015;212:502.e1-14.
39. Davenport MH, Ruchat SM, Giroux I, et al. Timing of excessive pregnancy-related weight gain and offspring adiposity at birth. *Obstet Gynecol* 2013;122:255e61.
40. Gaillard R, Steegers EA, Franco OH, et al. Maternal weight gain in different periods of pregnancy and childhood cardio-metabolic outcomes. The Generation R Study. *Int J Obes (Lond)* 2015;39:677-85.
41. Ruchat SM, Allard C, Doyon M, et al. Timing of Excessive Weight Gain During Pregnancy Modulates Newborn Anthropometry. *J Obstet Gynaecol Can* 2016;38:108-17.
42. Pitkin RM. Nutritional support in obstetrics and gynecology. *Clin Obstet Gynecol* 1976;19:489-513.
43. Nelson SM, Matthews P, Poston L. Maternal metabolism and obesity: modifiable determinants of pregnancy outcome. *Hum Reprod Update* 2010;16:255-75.
44. Hickey CA, Cliver SP, Goldenberg RL, et al. Relationship of psychosocial status to low prenatal weight gain among nonobese black and white women delivering at term. *Obstet Gynecol* 1995;86:177-183.
45. Olson CM, Strawderman MS. Modifiable behavioral factors in a biopsychosocial model predict inadequate and excessive gestational weight gain. *J Am Diet Assoc* 2003;103:48-54.
46. Brawarsky P, Stotland NE, Jackson RA, et al. Pre-pregnancy and pregnancy-related factors and the risk of excessive or inadequate gestational weight gain. *Int J Gynaecol Obstet* 2005;91:125-131.
47. Perng W, Gillman MW, Fleisch AF, et al. Metabolomic profiles and childhood obesity. *Obesity (Silver Spring)* 2014;22:2570-8.

For peer review only - http://bmjopen.bmj.com/site/about/guidelines.xhtml

48. Tyrrell J, Richmond RC, Palmer TM. Genetic Evidence for Causal Relationships between Maternal Obesity-Related Traits and Birth Weight. *JAMA* 2016;315:1129-40.
49. Ngo TTM, Moufarrej MN, Rasmussen MH et al. Noninvasive blood tests for fetal development predict gestational age and preterm delivery. Science 2018; 360 (6393): 1133-1136.
50. Santos Ferreira DL, Williams DM, Kangas AJ, et al. Association of pre-pregnancy body mass index with offspring metabolic profile: Analyses of 3 European prospective birth cohorts. *PLoS Med* 2017;14:e1002376.
51. Patel N, Hellmuth C, Uhl O. Cord Metabolic Profiles in Obese Pregnant Women: Insights Into Offspring Growth and Body Composition. *J Clin Endocrinol Metab* 2018;103:346-355.
52. Scholtens DM, Bain JR, Reisetter AC, et al. Metabolic Networks and Metabolites Underlie Associations Between Maternal Glucose During Pregnancy and Newborn Size at Birth. *Diabetes* 2016;65:2039-50.
53. Freinkel N. Banting Lecture 1980. Of pregnancy and progeny. *Diabetes*1980;29:1023–1035.
54. Census of India, 2011, Rajasthan, http://www.rajcensus.gov.in/).
55. Annual Health Survey (2011-12). Vital Statistics Division Office of the Registrar General & Census Commissioner, India New Delhi.
56. Ciborowski M, Zbucka-Kretowska M, Bomba-Opon D, et al. Potential first trimester metabolomic biomarkers of abnormal birth weight in healthy pregnancies. *Prenat Diagn* 2014;34:870-7.
57. Maitre L, Fthenou E, Athersuch T, et al. Urinary metabolic profiles in early pregnancy are associated with preterm birth and fetal growth restriction in the Rhea mother-child cohort study. *BMC Med* 2014;12:110.
58. Kenny LC, Broadhurst DI, Dunn W, et al. Robust Early Pregnancy Prediction of Later Preeclampsia Using Metabolomic Biomarkers. *Hypertension* 2010;56:741−49.
59. Zheng X, Su M, Pei L, et al. Metabolic signature of pregnant women with neural tube defects in offspring. *J. Proteome Res* 2011;10:4845−54.
60. Bahado-Singh RO, Akolekar R, Mandal R, et al. First-trimester metabolomic detection of late-onset preeclampsia. *Am J Obstet Gynecol* 2013;208:58.e1−7.
61. Paine MA, Scioscia M, Williams PJ, et al. Urinary inositol phosphoglycan P-type as a marker for prediction of preeclampsia and novel implications for the pathophysiology of this disorder. *Hypertens Pregnancy* 2010;29:375–84.
62. Dessi A, Atzori L, Noto A, et al. Metabolomics in newborns with intrauterine growth retardation (IUGR): urine reveals markers of metabolic syndrome. *J Matern Fetal Neonatal Med* 2011;2:35-9.
63. Ivorra C, García-Vicent C, Chaves FJ, et al. Metabolomic profiling in blood from umbilical cords of low birth weight newborns. *J Transl Med* 2012;10:142.

For peer review only - http://bmjopen.bmj.com/site/about/guidelines.xhtml

64. Walsh JM, Wallace M, Brennan L, et al. Early pregnancy maternal urinary metabolomic profile and later insulin resistance and fetal adiposity. *J Matern Fetal Neonatal Med* 2015;28:1697-700.
65. Tzoulaki I, Ebbels TM, Valdes A, et al. Design and analysis of metabolomics studies in epidemiologic research: a primer on – omic technologies. *Am J Epidemiol* 2014;180:129-39.
66. Tuulia Tynkkynen T, Wang Q, Ekholm J, Anufrieva O et al. Proof of concept for quantitative urine NMR metabolomics pipeline for large-scale epidemiology and genetics. **BioRxiv**. doi: https://doi.org/10.1101/288993
67. Pinto J, Barros AS, Domingues MR, et al. Following healthy pregnancy by NMR metabolomics of plasma and correlation to urine. *J Proteome Res* 2015;14:1263-74.
68. Odegård RA, Vatten LJ, Nilsen ST, et al. Preeclampsia and fetal growth. *Obstet Gynecol* 2000;96:950-5.
69. Liu F, Zhao C, Liu L, et al. Peptidome profiling of umbilical cord plasma associated with gestational diabetes-induced fetal macrosomia. *J Proteomics* 2016;139:38-44.
70. Lampl M, Kusanovic JP, Erez O, et al. Early rapid growth, early birth: accelerated fetal growth and spontaneous late preterm birth. *Am J Hum Biol* 2009;21:141-50.
71. Lee AC, Katz J, Blencowe H, et al. National and regional estimates of term and preterm babies born small for gestational age in 138 low-income and middle-income countries in 2010. *Lancet Glob Health* 2013;1:e26-36.
72. Ebrahim S, Kinra S, Bowen L, et al. The effect of rural-to-urban migration on obesity and diabetes in India: a cross-sectional study. *PLoS Med* 2010;7:e1000268.
73. Hagströmer M, Oja P, Sjöström M. The International Physical Activity Questionnaire (IPAQ): A study of concurrent and construct validity. *Public Health Nutr* 2006;9:755-62.
74. Bertolotto A, Volpe L, Calianno A, et al. Physical activity and dietary habits during pregnancy: effects on glucose tolerance. *J Matern Fetal Neonatal Med* 2010;23:1310-4.
75. Harizopoulou VC, Kritikos A, Papanikolaou Z, et al. Maternal physical activity before and during early pregnancy as a risk factor for gestational diabetes mellitus. *Acta Diabetol* 2010;47:83-9.
76. International Institute for Population Sciences (IIPS) and ICF. 2017. National Family Health Survey (NFHS-4), 2015-16: India. Mumbai: IIPS. http://www.rchiips.org/nfhs/nfhs4.shtml.
77. Poongothai S, Pradeepa R, Ganesan A, et al. Reliability and validity of a modified PHQ-9 item inventory (PHQ-12) as a screening instrument for assessing depression in Asian Indians (CURES-65). *J Assoc Physicians India* 2009;57:147-52.

For peer review only - http://bmjopen.bmj.com/site/about/guidelines.xhtml

78. Shivalli S, Gururaj N. Postnatal depression among rural women in South India: do socio-demographic, obstetric and pregnancy outcome have a role to play? *PLoS One* 2015;10:e0122079.
79. Topp CW, Østergaard SD, Søndergaard S, et al. The WHO-5 Well-Being Index: a systematic review of the literature. *Psychother Psychosom* 2015;84:167-76.
80. Al-Janabi H, Flynn TN, Coast J. Development of a self-report measure of capability wellbeing for adults: the ICECAP-A. *Qual Life Res* 2012;21:167-76.
81. Ilias Goranitis, Joanna Coast, Hareth Al-Janabi, et al. The validity and responsiveness of the ICECAP-A capability-well-being measure in women with irritative lower urinary tract symptoms. *Qual Life Res* 2016;25:2063–2075.
82. Keeley T, Al-Janabi H, Nicholls E, et al. A longitudinal assessment of the responsiveness of the ICECAP-A in a randomised controlled trial of a knee pain intervention. *Qual Life Res.* 2015;24:2319–2331.
83. Villar J, Altman DG, Purwar M, et al. The objectives, design and implementation of the INTERGROWTH-21st Project. *BJOG* 2013; 120:9-26.
84. Eskenazi B, Bradman A, Finkton D, et al. A rapid questionnaire assessment of environmental exposures to pregnant women in the INTERGROWTH 21st Project. *BJOG* 2013;120:129-38,v.
85. The International Society for the Advancement of Kinanthropometry. *International Standards for Anthropometric Assessment*. Australia: ISAK, 2001.
86. Cheikh Ismail L, Knight HE, Bhutta Z, et al. International Fetal and Newborn Growth Consortium for the 21st Century. Anthropometric protocols for the construction of new international fetal and newborn growth standards: the INTERGROWTH-21st Project. *BJOG* 2013;120:42-7, v.
87. Illig T, Gieger C, Zhai G, et al. A genome-wide perspective of genetic variation in human metabolism. *Nat Genet* 2010;42:137-41.
88. Altmaier E, Emeny RT, Krumsiek J, et al. Metabolomic profiles in individuals with negative affectivity and social inhibition: a population-based study of Type D personality. *Psychoneuroendocrinology* 2013;38:1299-309.
89. Breier M, Wahl S, Prehn C. Targeted metabolomics identifies reliable and stable metabolites in human serum and plasma samples. *PLoS One* 2014;9:e89728.
90. Petersen AK, Zeilinger S, Kastenmüller G, et al. Epigenetics meets metabolomics: an epigenome-wide association study with blood serum metabolic traits. *Hum Mol Genet* 2014;23:534-45.
91. Johnson CH, Gonzalez FJ. Challenges and opportunities of metabolomics. *J Cell Physiol* 2012;227:2975-81.
92. Suhre K, Shin SY, Petersen AK, et al. Human metabolic individuality in biomedical and pharmaceutical research. *Nature* 2011;477:54-60.

For peer review only - http://bmjopen.bmj.com/site/about/guidelines.xhtml

93. Liao SG, Lin Y, Kang DD, et al. Missing value imputation in high-dimensional phenomic data: imputable or not, and how? *BMC Bioinformatics* 2014;15:346.
94. Papageorghiou AT, Kennedy SH, Salomon LJ, et al. International Fetal and Newborn Growth Consortium for the 21st Century (INTERGROWTH-21st). International standards for early fetal size and pregnancy dating based on ultrasound measurement of crown-rump length in the firts trimester of pregnancy. *Ultrsound Obstet Gyneco* 2014;44:641-8.
95. Stirnemann J, Villar J, Salomon LJ, et al. International estimated fetal weight standards of the INTERGROWTH-21st Project. *Ultrasound Obstet Gynecol* 2017;49:478-486.
96. National Institute of Health and Care Excellence (NICE), National Collaborating Centre for Women's and Children's Health. NICE guideline: antenatal care. London: Royal College of Obstetricians and Gynaecologists Press, 2008.
97. Eichenberger Gilmore JM, Pauley CA, Burns TL, et al. A hip analysis protocol for pediatric bone desnsitometry: the Iowa Bne Development Study. *J Clin Densitom* 2010;13:361-9.
98. International Atomic Energy Agency, 2013. Body composition assessment from birth to 2 years of age. Vienna, IAEA human health series ISSN 2075-3772 no-22.
99. Shepherd JA, Sommer MJ, Fan B, et al. Advanced Analysis Techniques Improve Infant Bone and Body Composition Measures by Dual-Energy X-Ray Absorptiometry. *J Pediatr* 2017;181:248-253.e3.
100. Cheikh Ismail L, Bishop DC, Pang R, et al. Gestational weight gain standards based on women enrolled in the Fetal Growth Longitudinal Study of the INTERGROWTH-21st Project: a prospective longitudinal cohort study. *BMJ* 2016;352:i555.
101. Nakai M, Ke W. Statistical Models for Longitudinal Data Analysis. *Applied Mathematical Sciences* 2009;3:1979–89.
102. Baron RM, Kenny DA. The moderator-mediator variable distinction in social psychological research: conceptual, strategic, and statistical considerations. *J Pers Soc Psychol* 1986;51:1173–82.
103. Storey JD. The positive false discovery rate: A Bayesian interpretation and the q-value. *The Annals of Statistics* 2003;31:2013–35.
104. Razak F, Corsi DJ, Subramanian SV. Change in the body mass index distribution for women: analysis of surveys from 37 low- and middle-income countries. *PLoS Med* 2013;10:e1001367.
105. Jones AD, Hayter AK, Baker CP. The co-occurrence of anemia and cardiometabolic disease risk demonstrates sex-specific socio demographic patterning in an urbanizing rural region of southern India. *Eur J Clin Nutr* 2016;70:364-72.
106. Mithal A, Bansal B, Kalra S. Gestational diabetes in India: Science and society. Indian *J Endocrinol Metab* 2015;19:701-4.

For peer review only - http://bmjopen.bmj.com/site/about/guidelines.xhtml

107. Agrawal S, Walia GK. Prevalence and Risk Factors for Symptoms Suggestive of PreEclampsia in Indian Women. *Journal of Womens Health Issues Care* 2014;3:6.
108. Sharma R. Birth defects in India: Hidden truth, need for urgent attention. *Indian J Hum Genet* 2013;19:125-9.
109. Bhati DK. Stillbirths: A high magnitude public health issue in India. *South East Asia Journal Of Public Health* 2013;3:3-9.
110. Subramanyam MA, Kawachi I, Berkman LF, et al. Is economic growth associated with reduction in child undernutrition in India? *PLoS Med* 2011;8:e1000424.
111. Metzger BE. Long-term outcomes in mothers diagnosed with gestational diabetes mellitus and their offspring. *Clin Obstet Gynecol* 2007;50:972–979.
112. Dabelea D, Crume T. Maternal environment and the transgenerational cycle of obesity and diabetes. *Diabetes* 2011;60:1849–1855.
113. Catalano PM, Hauguel-De Mouzon S. Is it time to revisit the Pedersen hypothesis in the face of the obesity epidemic? *Am J Obstet Gynecol* 2011;204:479–487.

For peer review only

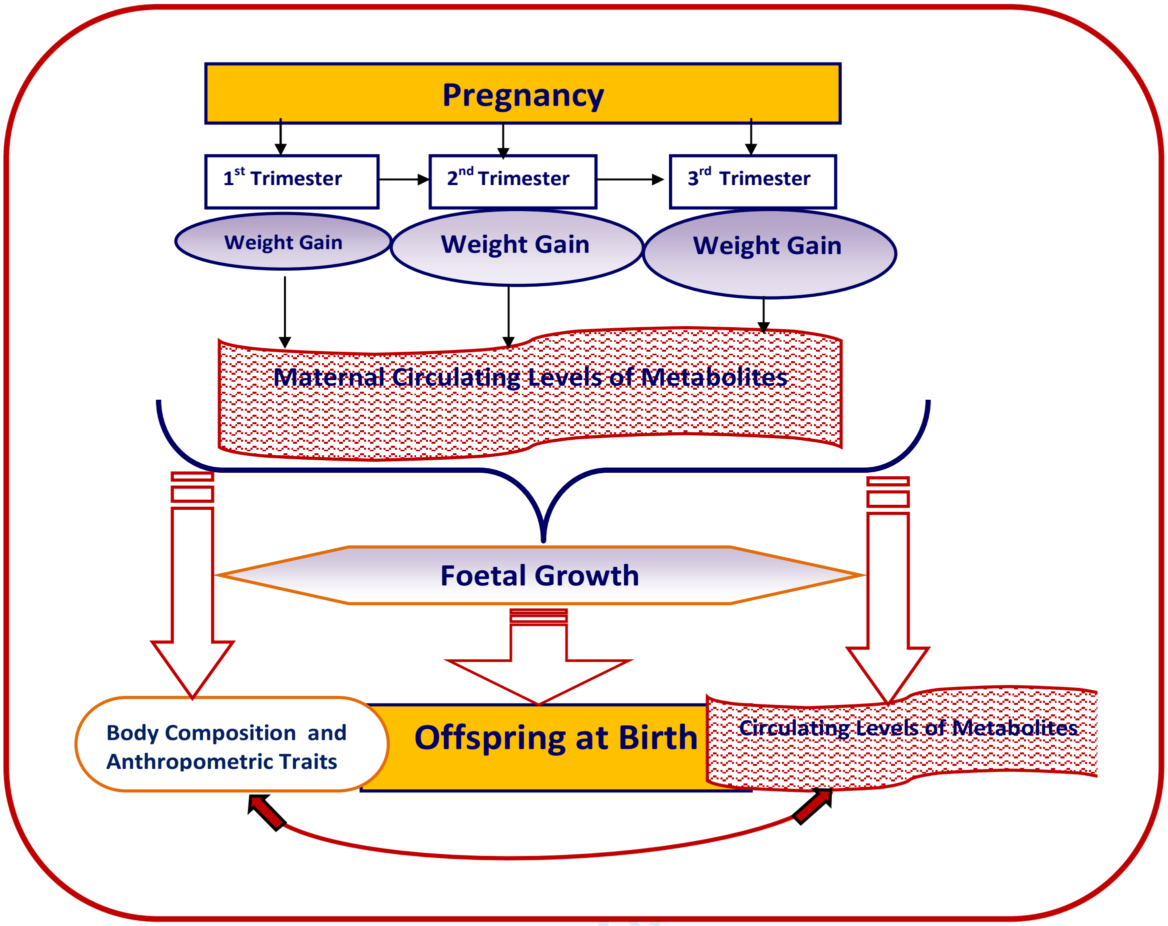

Figure-1: Diagrammatic representation of hypothesis: *Quantitative changes in maternal circulating metabolites associated with gestational weight gain, influences foetal growth, offspring's levels of metabolites, body composition and anthropometric traits at birth.*

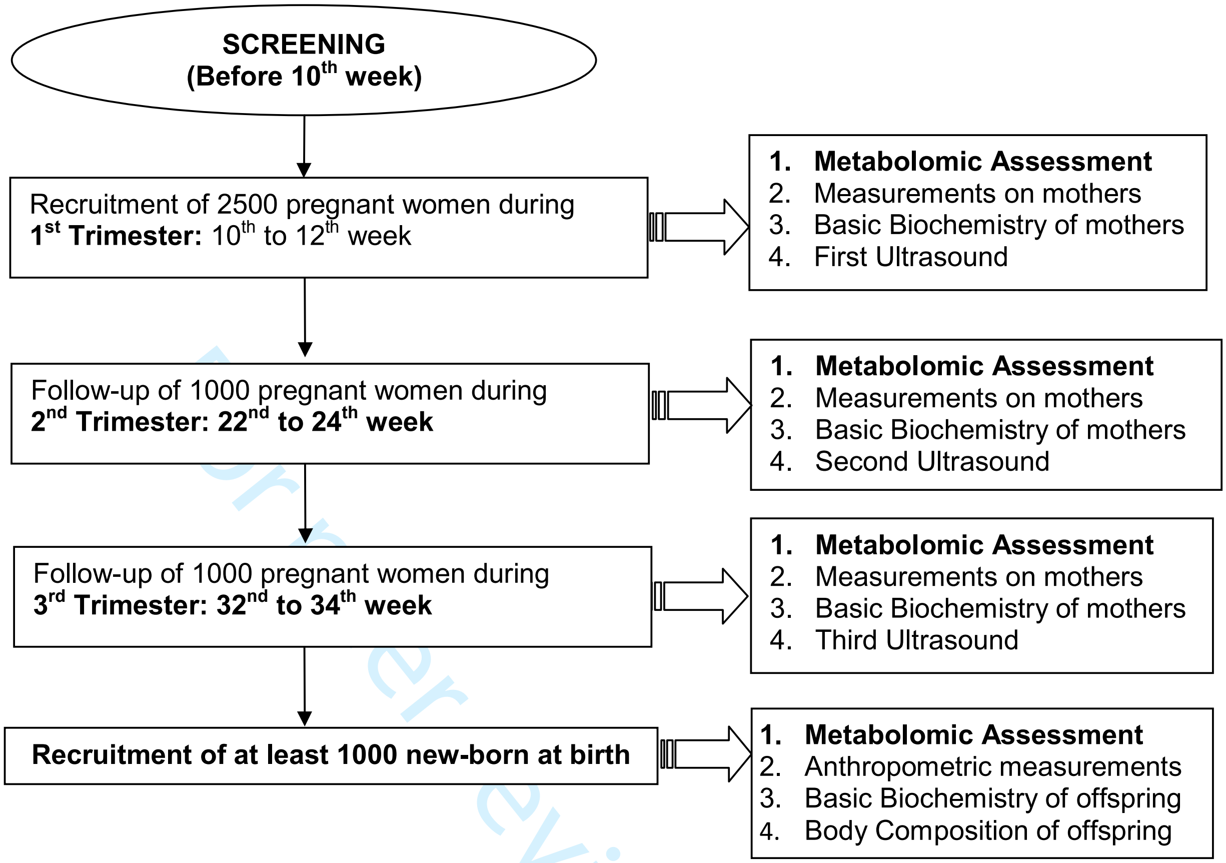

Figure-2: Recruitment and follow-up strategy

For peer review only

Gestational Route to Healthy Birth (GaRBH): protocol for an Indian prospective cohort study

Journal:	*BMJ Open*
Manuscript ID	bmjopen-2018-025395.R1
Article Type:	Protocol
Date Submitted by the Author:	17-Oct-2018
Complete List of Authors:	Gupta, Vipin; University of Delhi, Department of Anthropology<br>Saxena, Ruchi; Sardar Patel Medical College, Department of Obstetrics and Gynaecology<br>Walia, Gagandeep; Public Health Foundation of India<br>Agarwal, Tripti; Public Health Foundation of India<br>Vats, Harsh; Department of Anthropology, University of Delhi<br>Dunn, Warwick; School of Biosciences, Phenome Centre Birmingham and Institute of Metabolism and Systems Research, University of Birmingham, UK<br>Relton, Caroline; MRC Integrative Epidemiology Unit and Bristol Medical School, University of Bristol, UK<br>Sovio, Ulla; University of Cambridge, Obstetrics and Gyneacology<br>Papageorghiou, Aris; University of Oxford<br>Davey Smith, George; MRC Integrative Epidemiology Unit and Bristol Medical School, University of Bristol, UK<br>Khadgawat, Rajesh; All India Institute of Medical Sciences, New Delhi<br>Sachdeva, Mohinder; Department of Anthropology, University of Delhi
**Primary Subject Heading**:	Epidemiology
Secondary Subject Heading:	Public health, Nutrition and metabolism
Keywords:	Birth cohort, India, Developmental origin, Foetal growth, Metabolomics in Pregnancy, Epidemiology < TROPICAL MEDICINE

Title:

Gestational Route to Healthy Birth (GaRBH): protocol for an Indian prospective cohort study

Vipin Gupta[1#], Ruchi Saxena[2#], Gagandeep Kaur Walia[3#], Tripti Agarwal[3], Harsh Vats[1], Warwick Dunn[4], Caroline Relton[5], Ulla Sovio[6], Aris T. Papageorghiou[7], George Davey Smith[5], Rajesh Khadgawat[8] and M. P. Sachdeva[1]

[1]Department of Anthropology, University of Delhi, India
[2]Sardar Patel Medical College, Bikaner, Rajasthan, India
[3]Public Health Foundation of India, India
[4]School of Biosciences, Phenome Centre Birmingham and Institute of Metabolism and Systems Research, University of Birmingham, UK
[5] MRC Integrative Epidemiology Unit and Bristol Medical School, University of Bristol, UK
[6]Department of Obstetrics and Gynaecology, School of Clinical Medicine, University of Cambridge, UK
[7]Nuffield Department of Women's and Reproductive Health, University of Oxford, UK
[8]Department of Endocrinology, Metabolism and Diabetes, All India Institute of Medical Sciences, India
[#]Authors contributed equally

Corresponding Author

Dr. Vipin Gupta
Department of Anthropology,
University of Delhi, Delhi-110007, India
Email: udaiig@gmail.com
Mobile: 9899119242

For peer review only

Abstract

Introduction: Pregnancy is characterized by a high rate of metabolic shifts from early to late phases of gestation in order to meet the raised physiological and metabolic needs. This change in levels of metabolites is influenced by gestational weight gain (GWG) which is an important characteristic of healthy pregnancy. Inadequate/excessive GWG has short and long term implications on maternal and child health. Exploration of gestational metabolism is required for understanding the quantitative changes in metabolite levels during the course of pregnancy. Therefore, our aim is to study trimester-specific variation in levels of metabolites in relation to GWG and its influence on foetal growth and newborn anthropometric traits at birth.

Methods and analysis: A prospective longitudinal study is planned (start date: February, 2018; end date: March, 2023) on pregnant women that are being recruited in the first trimester and followed in subsequent trimesters and at the time of delivery (total 3-follow-ups). The study is being conducted in a hospital located in Bikaner district (66% rural population), Rajasthan, India. The estimated sample size is of 1000 mother-offspring pairs. Information on gynaecological and obstetric history, socioeconomic position, diet, physical activity, tobacco and alcohol consumption, depression, anthropometric measurements and blood samples is being collected for metabolic assays in each trimester using standardized methods. Mixed effects regression models will be used to assess the role of gestational weight in influencing metabolite levels in each trimester. The association of maternal levels of metabolites with foetal growth, offspring's weight and body composition at birth will be investigated using regression modelling.

Ethics and dissemination: The study has been approved by the ethics committees of the Department of Anthropology, University of Delhi and Sardar Patel Medical College, Rajasthan. We are taking written informed consent after discussing the various aspects of the study with the participants in the local language.

Key words: Birth cohort, India, Metabolomics in Pregnancy, Foetal growth, Epidemiology and Developmental origin

Word Count: 5521

For peer review only - http://bmjopen.bmj.com/site/about/guidelines.xhtml

Article Summary

Strength and Limitations

- This is a longitudinal study in India to examine trimester specific metabolomic changes during the course of pregnancy and their influence on foetal growth.
- The study is collecting biological measurements on pregnant women during each trimester (1st, 2nd and 3rd trimester) and their offspring at the time of birth.
- Newborns will be intensively phenotyped for the levels of metabolites and body composition.
- Unlike other Indian cohorts, the study participants are being recruited from a government hospital that caters for a rural population.
- Lack of information on pre-pregnancy measurements will restrict our analysis to the duration of pregnancy only.

For peer review only - http://bmjopen.bmj.com/site/about/guidelines.xhtml

Introduction

The profound metabolic changes during healthy pregnancy ensure optimum foetal development and appropriate nutrient allocation between the mother and foetus.[1,2] Such metabolic adjustments are required for meeting raised physiological needs of pregnancy and for provision of additional energy to support labour and lactation.[1] The concept of *"the developmental origins of health and disease"* suggests that exposures during critical periods *in utero* can lead to *"programmed"* structural and/or functional alterations that can transmit to the next generation and predispose them to disease at later age.[3,4] A study on 115 women with normal pregnancy has found that higher circulating fatty acids are associated with lower blood pressure and more favourable metabolic phenotype of the mothers.[5] The disparity in metabolic changes during pregnancy may influence foetal growth, and can increase the likelihood of adverse metabolic profiles across the life-course for both mother and the offspring through foetal programming.[6] For instance, increased transport of glucose and free fatty acids to the foetal-placental unit can lead to adverse foetal growth (i.e. macrosomia).[7,8]

The biological system-wide understanding of metabolism requires us to capture phenotypic changes of the human *metabolome,* which comprises the full spectrum of metabolites present in all cells, tissues or fluids of the human body,[9,10,11] in different physiological states (i.e. puberty, pregnancy, lactation and pre/post-menopause). The metabolome can be considered as an intermediate phenotypic mixture of metabolites encoded by our genome, influenced by specific environmental exposures, and it shapes an individual's *metabolic phenotype or* metabotype.[10,11,12] It can be studied through the holistic approach of *metabolomics* that provides a quantitative fingerprint of all metabolites present in cells, tissues or an organism and can be measured using high throughput technologies.[13,14] The field of metabolomics is important for uncovering quantitative metabolic trajectories of normal and abnormal pregnancies. The studies on quantitative estimation of longitudinal metabolomic changes during gestation are globally few in number[15,16,17] and there are none we know of in India.

For peer review only - http://bmjopen.bmj.com/site/about/guidelines.xhtml

Metabolomic changes in pregnancy

The incomplete understanding of the complex nature of metabolic processes during pregnancy has generated considerable interest in exploring the effect of gestational characteristics on metabolomic changes during pregnancy and its impact on offspring health. Hyperglycemia during pregnancy is one of the widely studied examples of pregnancy induced metabolic stress which increases maternal risk of type 2 diabetes and also predisposes the developing foetus to poor metabolic health.[18] Studies have also assessed the metabolomics of pregnancy complications, such as, gestational diabetes,[19,20] placental abruption[21], and birth outcomes, such as preterm birth[22], small for gestational age[23] and large for gestational age.[24]

An exploratory study has investigated the changes in metabolite levels during pregnancy among women with gestational diabetes in the third trimester and found elevated levels of branched chain amino acids, leucine, isoleucine, and valine.[25] The catabolic nature of third trimester is represented by a decrease in maternal glucose levels and substantial rise in the levels of fasting insulin which in turn can lead to insulin resistance.[26] Moreover, circulating levels of lipids and lipoproteins rise 2-3 fold in late pregnancy.[27] Maternal amino acid levels change longitudinally due to placental uptake and foetal transfer from 1st to 3rd trimesters.[15] There is an increase in energy production through the TCA cycle during the course of gestation along with concomitant decreases in free carnitine and acetylcarnitine and increase in carnitine palmitoyltransferase-1 activity and β-hydroxybutyrate levels.[15] A recent study has detected a role for metabolites in foetal sex differences in placental complications. It was found that higher maternal levels of spermine metabolite N1,N12-diacetylspermine (DiAcSpm), from the 1st to 3rd trimesters, are observed in the female placenta and in the serum of women with a female foetus.[17]

Relationships between maternal gestational weight gain and newborn health

Gestational weight gain (GWG) is a complex biological trait which is influenced by maternal physiology, maternal, placental and foetal metabolism.[28] There are short or long term implications of inadequate or excessive GWG which direct the trade-off between maternal and child health outcomes.[28,29,30,31.32] For example, inadequate weight gain is associated with high risks of low birth weight, small for gestational age, and preterm birth.[33] Excessive GWG is associated with large for gestational age, gestational diabetes, caesarean section and postpartum weight retention.[34] Further, high pre-pregnancy maternal body mass index (BMI) is associated with perturbations to maternal metabolome across gestation.[35]

Maternal GWG is a good indicator of foetal nutrition.[36] Any amount of weight gain in the first 14 weeks and high rate of weight gain (>500g/week) in 14 to 36 weeks are

For peer review only - http://bmjopen.bmj.com/site/about/guidelines.xhtml

associated with raised adiposity in the offspring.[37] GWG in the first trimester is associated with offspring BMI from infancy to early childhood whereas excessive GWG (i.e. ≥500g/week) in 2nd and 3rd trimesters is associated with increased birth weight.[38] In the first half of the pregnancy (<20 weeks), excessive GWG is associated with higher birth weight and high body fat in newborns compared to the second half of pregnancy.[39] Further, Gaillard et al.[40] observed that early GWG was associated with increased adiposity levels and adverse cardio-metabolic profile in childhood independent of pre-pregnancy weight. A study that investigated the effect of trimester specific GWG on newborn anthropometry has found that women with an excessive rate of GWG in mid/late pregnancy deliver the heaviest babies.[41] Thus, the role of optimum GWG in foetal and child health is well studied[42] but the underlying biological mechanisms or mediating pathways are as yet unknown. The maternal fat deposition in early pregnancy due to excessive GWG may lead to maternal dysmetabolism[43,44] and effect the levels of maternal metabolites in subsequent trimesters. A study has found that the levels of triglycerides, HDL-C, apoA1 and IL-6 are linearly associated with GWG across all levels of GWG in mid pregnancy.[37] The authors postulated that mothers with high GWG may engage in lifestyles during and after pregnancy that may encourage weight gain. However, a few Indian studies have incorporated the GWG in their analyses, for example, an association of inadequate or excessive GWG with pre-term deliveries[45], average GWG among women with gestational diabetes[46], and maternal dietary intake of milk protein in first trimester on GWG[47]. But there is no Indian study that has explored the association between GWG and levels of metabolites. The continuous variability in the amount of weight gain during pregnancy, with respect to parity, height and geography,[48,49,50] has motivated the research for detecting the predictors and outcomes of GWG.

Maternal metabolic associations with newborn phenotypes

Maternal pre-pregnancy obesity can predict higher branched chain amino acids and related metabolites in offspring.[51] The increase in maternal pre-pregnancy BMI is strongly associated with adverse cardio-metabolic profile (very-low-density lipoprotein (VLDL)-lipoproteins, VLDL cholesterol, VLDL-triglycerides, VLDL-diameter, branched/aromatic amino acids, glycoprotein acetyls, and triglycerides) in offspring.[52] A Mendelian randomization study has suggested causal relationships between genetically elevated maternal pre-pregnancy BMI (1 standard deviation) and blood glucose levels (7.2mg/dl) with higher birth weight [55g (95%CI: 93g; p-value: 0.004) and 114g (95%CI: 147g; p-value: $7x10^{-9}$), respectively] of the offspring.[53] The metabolic shift from first to third trimester of gestation can influence newborn phenotypes. A large study related to the effects of maternal metabolic signatures on foetal growth has identified urinary metabolites (like branched-chain amino acids, isoleucine, valine, leucine, alanine,

For peer review only - http://bmjopen.bmj.com/site/about/guidelines.xhtml

choline, 3 hydroxyisobutyrate, progesterone and estrogens) predictive of foetal growth, and explained 48-53% of total variation in birth weight.[16] Recently, a pilot study has shown that both gestational age of the pregnancy (comparable to that estimated by ultrasound) and risk for preterm delivery can be predicted by measuring cell-free RNA transcripts in maternal blood during pregnancy.[54] This study highlighted the opportunities offered in the identification of non-invasive blood based biomarkers of pregnancy outcome.[54] It has been shown that maternal hyperglycemia is associated with higher cord blood concentrations of lysophosphatidylcholines in offspring independent of lifestyle intervention among obese women.[55] Thus, maternal pre-pregnancy BMI, RNA transcripts and hyperglycemia during gestation influences foetal and newborn phenotypes.

Scholtens et al.[56] assessed the impact of maternal hyperglycemia, measured using an oral glucose tolerance test (OGTT), on foetal phenotypes at 28 weeks of gestation. They observed a decrease in amino acids, fatty acids, and products of lipid metabolism whereas a rise in triglycerides and carbohydrates between fasting and 1-hour OGTT samples. Further, the role of maternal glucose, triglycerides and fatty acids in predicting the newborn birth weight and sum of skinfolds thicknesses was also suggested. This confirmed the Freinkel modification of the Pedersen hypothesis i.e. foetal growth and fat accumulation in the setting of maternal hyperglycemia is contributed to by other nutrients in addition to glucose.[57] The longitudinal assessment of the role of maternal metabolites during the length of the pregnancy on birth outcomes is restricted to four studies, of which two studies utilize urine samples[16,58] and the remaining two blood samples.[15,17]

Gestational Route to Healthy Birth (GaRBH) cohort study

GWG in first trimester acts as an exposure for the levels of metabolites as an outcome in subsequent trimesters. Similarly, the levels of metabolites in a trimester can also act as an exposure for GWG in next trimester(s). Therefore, this study aims to quantify differential metabolic variability in relation to maternal weight gain during pregnancy for assessing its influence, as an exposure, on a) foetal growth, b) offspring metabolite levels, c) body composition of offspring at birth. The conceptual model is given in Figure-1. We are asking two research questions:

1. Are quantitative changes in levels of metabolites associated with gestational weight gain during pregnancy?
2. Are quantitative metabolic changes during pregnancy associated with foetal growth, offspring levels of metabolites and body composition at birth?

Study objectives

For peer review only - http://bmjopen.bmj.com/site/about/guidelines.xhtml

- To detect differential changes in quantitative metabolites between the end of the 1st, 2nd and 3rd trimesters of pregnancy and to examine how these trajectories are associated with maternal weight gain.
- To determine the influence of metabolic changes during pregnancy on foetal growth, body composition (fat mass, lean mass and bone density) and anthropometry at birth (i.e. body length, weight and circumferences) independent of maternal pre-pregnancy BMI and paternal phenotypic characteristics.
- To assess the association between maternal metabolic profile during pregnancy and metabolite levels of offspring at birth (in cord blood).

Insert Figure-1

For peer review only

Study area, design and setting

Study Area: This study is being conducted in the government hospital of Sardar Patel Medical College, i.e. "Prince Bijay Singh Memorial Hospital" (PBM), located in the sub-district *Bikaner* which is one of the eight sub-districts ("*Tehsils*") of Bikaner district in Rajasthan state. According to Census of India,[59] Rajasthan is India's largest state in terms of geographical area, with a population size of 68 million. The population of Bikaner district is 2.3 million (66% is rural) of which 47% are females. In Bikaner, crude birth rate is 26 per 1000 individuals, the total fertility rate is 3 and the infant mortality rate is 60 per 1000 individuals.[60] The majority of study participants are homemakers (80%), follow the Hindu religion (70%) and approximately 20% of the participants are illiterate. The choice of PBM hospital was based on the availability of government medical facility which is the largest in district Bikaner and it ensures accessibility and affordability (due to free of cost treatment) for the entire population.

Study Design: A hospital based cohort study which is recruiting and longitudinally following the pregnant women until delivery, and their offspring at birth in district *Bikaner,* Rajasthan, India.

Sample size: Studies of fairly large size for detecting associations between longitudinal changes in levels of metabolites in pregnancy and foetal growth, and levels of metabolites of the offspring at birth, are few in number. For example, Ciborowski et al.[61] used 770 samples collected during the first trimester for studying the relationship between maternal metabolic levels with macrosomia and low birth weight. Maitre et al.[58] investigated 34 metabolites on 438 samples recruited during the first trimester and found an association with foetal growth restriction and preterm birth. The majority of studies related to metabolic changes during pregnancy[62,63,64] and maternal-foetal medicine[65,66] were based on 30-60 samples.[67,68] Exact power calculations are challenging due to a restricted number of prior studies leading to unknown features.[69]

For peer review only - http://bmjopen.bmj.com/site/about/guidelines.xhtml

Even the largest study on 4212 participants for studying the predictive metabolomic biomarker for placental complications included limited sample of preeclampsia (n=134), foetal growth restriction (n=162) and controls (n=259).[17] Thus, the proposed sample size must be larger than the majority of informative studies conducted to date and feasible within the cost boundaries for the technology being used.

Tynkkynen et al.[70] studied quantitative urine and serum metabolite data on 995 samples of Northern Finland Birth Cohort for establishing the proof of concept for conducting large scale epidemiological studies in metabolomics using an example of BMI. A relatively large study (n=800) has found that maternal urine metabolites can independently explain 12% of birth weight after adjusting for all other lifestyle/ clinical factors.[16] They also found an association of trimester specific metabolic changes on foetal weight (change of 1.2% in first trimester and 2.4% in third trimester). Studies have observed that a metabolite level may change from 10 to 30% (even more for some metabolites) from first trimester to third trimester.[15,71]

For a cross-sectional analysis, a sample size of 1000 will be sufficient (with 98% power) to explain 4-5% variation in the level of a metabolite by maternal weight as a predictor, even after correcting for multiple testing (α= 0.0001; for number of metabolites). Moreover, a sample size of 1000 will also be sufficient to explain 2% variation in the foetal growth parameters (with 80% power, α= 0.0001) by maternal level of metabolite as a predictor.

Therefore, we propose to recruit 2500 pregnant women during the first trimester in order to measure metabolites on at least 1000 pregnant women (we may recruit >1000 women) in each trimester and their offspring, accounting for loss to follow-up.

Recruitment Strategy

Women visiting the antenatal clinic of the PBM hospital are being screened with the help of a one-page questionnaire to assess their eligibility for the study. We aim to screen around 6000 pregnant women from PBM hospital to reach the targeted sample size. The participants will have to satisfy the eligibility criteria (given below) before being recruited at baseline (Figure-2) from February, 2018 to March, 2023:

1. Less than or equal to 10 weeks of gestation
2. Age: ≥18 to <35 years
3. Singleton pregnancy
4. Natural conception (i.e. without any fertility treatment)
5. No long-term medication (i.e. not more than two weeks in last one year)
6. No more than one miscarriage in previous pregnancies. In cases where the current pregnancy is the second pregnancy, then the women with miscarriage in their first pregnancy will also be excluded.

For peer review only - http://bmjopen.bmj.com/site/about/guidelines.xhtml

7. No previous pregnancy affected by pre-eclampsia/eclampsia
8. No clinical evidence of sexually transmitted disease
9. No hormonal therapy

We will not exclude the study participants (recruited in first trimester) who develop pregnancy complications in subsequent trimesters that may influence foetal growth (like pre-eclampsia[72], eclampsia, intrauterine growth restriction and gestational diabetes mellitus[73]). Exclusion of these complications in current pregnancy would lead to a reduction in the natural variation in the study population. Further, we will not exclude preterm delivery cases due to its relationship with foetal growth[74] and its high burden (i.e. 12.8 million in 2010) in India[75], as it could lead to bias by restricting the sampling to very healthy pregnancies which may not represent the general population.

Insert Figure-2

Patient and public involvement

The fieldworkers are trained to establish rapport with the study population which begins with describing the research questions and objectives of the study to the pregnant women visiting the antenatal clinic of PBM hospital during first trimester. Once they agree to consider participation, project staff discusses the participant information sheet, containing detailed information on all the measures and outcomes of the project with them in local language. Our data collection process starts only after taking the informed written consent from the eligible participants. We constantly try to involve them during the pregnancy by providing the readings of biochemical parameters and ultrasounds in each trimester and facilitate their antenatal visits in PBM hospital. The project staff will also provide support to the participant during and after the childbirth in the hospital. Moreover, the study population will be engaged through public events for the final dissemination of the study results.

Assessment procedures and instrumentation

Ultrasound Measures

For ultrasound-based measurements relating to foetal growth, we are using the existing standard operating procedures described by INTERGROWTH study[76] to ensure reliable data collection. The data is being collected by trained sonographers of PBM hospital, Bikaner, who are highly familiar with the equipment (ALOKA Prosound Alpha-6, Hitachi Aloka Medical Systems, Tokyo, Japan) and experienced in conducting ultrasound examinations. The intra-observer variation will be assessed by repeat sonographic measurements on 10 participants on the same day. Comparisons will also

For peer review only - http://bmjopen.bmj.com/site/about/guidelines.xhtml

be made between the readings of two sonographers of the hospital on 10 participants to assess inter-observer variability. The inter-equipment variation in ultrasound measures will be assessed by comparing the PBM results of 10 participants with the radiology laboratory outside the hospital. We will measure the crown-rump length (CRL), biparietal diameter (BPD), occipito-frontal diameter (OFD), head circumference (HC), femur length (FL), anterior-posterior abdominal diameter (APAD). Gestational age based on CRL and foetal weight will be estimated with the help of INTERGROWTH equations.[76,77]

Estimation of Gestational Age

The accurate estimation of individual level gestational age (GA) during pregnancy is vital for interpreting foetal anatomy and growth patterns and predicting the date of delivery. GA is being calculated from the first day of the last menstrual period (LMP). Moreover, for women providing clear information on LMP (in completed weeks and days) their gestational age is being confirmed with an ultrasound based measurement of crown–rump length (CRL) in the first trimester, using an internationally recognized chart.[76] If the difference between the CRL and LMP estimates is ≤7 days, LMP is being considered valid and taken as the true biological date.[76] In women with a LMP and CRL difference of >7 days, CRL is being considered valid for estimating the gestational age.[78]

Questionnaires

A comprehensive questionnaire has been developed for collecting information on demographic, anthropological (caste, religion and consanguinity), medical disease history, tobacco and alcohol intake, and passive smoking. The validated food frequency questionnaire of "*Indian Migration Study*" is being used for evaluating dietary intake.[79] Maternal physical activity during pregnancy is being assessed using the international physical activity questionnaire (i.e. 7days long).[80,81,82] The assets based tool of National Family Health Survey-4 is being used to evaluate socioeconomic position.[83] For assessing depression, we are using the Patient Health Questionnaire[84] and Edinburgh Postnatal Depression scale.[85] The WHO-5 questionnaire is being used for collecting information on quality of life.[86] The capability wellbeing, i.e. individual's freedom to function in five key areas of their life: ''stability'', ''attachment'', ''achievement'', ''autonomy'' and ''enjoyment'', is being assessed by using the ICEpop CAPability measure for Adults (ICECAP-A).[87,88,89] Information on maternal environment, gynaecological and obstetrics history is also being collected using tools adapted from INTERGROWTH study.[90,91] Additionally, a structured questionnaire on social factors like relationships with family members, husband position in household, social support during

For peer review only - http://bmjopen.bmj.com/site/about/guidelines.xhtml

pregnancy, and travel is being used for assessing the quality of social life and adequacy of social support (Table-1).

Insert Table-1

Adult Anthropometry

The assessment of anthropometric traits (weight, height, sitting height, circumferences and skinfolds) are being performed on study participants using standard procedures.[92]

Weight is being measured when the participant standing straight on the centre of the weighing machine (Tanita, model-HD380), arms hanging freely with minimum clothes and without accessories. Height is being measured as the vertical distance between vertex (highest point on head) and floor when the participant standing straight with feet together on the level floor without footwear, arms resting by the side, with head in eye-ear plane (Frankfort horizontal plane), and heels, buttocks and shoulders projected on the vertical plane using a wall mounted stadiometer (ADE, Germany, model-MZ10023-3). Sitting height is being measured as the maximum distance from the vertex (top of the head) to the base of a flat sitting surface, with legs hanging freely and head in eye-ear plane.

Circumferences are being measured at the specific landmarks when the plane of the tape (ADE, Germany, model-MZ10021) is perpendicular to the long axis of the body and parallel to the floor without tape being pulled too loose or too tight. Waist circumference is being measured at the narrowest point (which is an approximate midpoint) around the waist between the lower rib margin of the last palpable rib and the top of the iliac crest with the help of a measuring tape. Mid-upper arm circumference is being measured at the mid-point between the tip of the shoulder and the tip of the elbow (acromion and olecranon process, respectively) taken horizontally (at the middle of the biceps). Hip circumference is being measured at the widest part of the hip (buttock) at the level of the trochanterion which is the most superior point on the greater trochanter of the femur, not the most lateral point. Calf circumference is being measured on the right leg as the maximum circumference in a plane perpendicular to the long axis of the calf until the greatest circumference is located when foot is placed on raised platform.

Skinfold measurements are being taken in relaxing muscles by grasping a distinct fold that separates from underlying muscles with the help of Harpenden Skinfold calliper when the jaws of the calliper are perpendicular to the length of the fold on the site of measurement. Skinfold measurements at biceps, triceps, sub-scapular, supra-iliac, medial calf and anterior thigh are being taken to the nearest 0.1mm.

For peer review only - http://bmjopen.bmj.com/site/about/guidelines.xhtml

Three readings are being taken for all the anthropometric measures with minimum acceptable difference of 100g, 5mm, 5mm and 2mm between the readings for weight, height, circumferences and skinfolds, respectively. The readings are being recorded to the last completed grams or millimetre.

Infant anthropometry

We will use the protocol of the International Fetal and Newborn Growth Consortium[93] for measuring the infants. Birth weight will be taken within 30 minutes of delivery, without clothes and in stable condition when the infant is placed an electronic weighing scale (ADE, model-M114600, Germany) to the nearest 5g. Infant length will be measured within 12 hours of birth using the Baby length measuring board (ADE range 100–1000 mm, Germany), which has a fixed headboard and moveable footboard, placed on a raised flat platform on the level surface. The head of the infant will be positioned in eye-ear plane on the board and holding the infant's legs with the left hand and moving the footboard with the right hand. Head circumference will be measured when the infant is on the lap of the field investigator, with stable head, using a measuring tape (SECA, 212) anchored just above the eyebrows on the forehead, and at the back the tape being positioned over the fullest protuberance of the skull. Three readings will be taken for all the newborn anthropometric traits with minimum acceptable difference of 10g, 5mm, and 2mm between the readings for weight, infant length and circumferences, respectively. The readings will be recorded to the last completed millimetre.

All the equipment used for the assessment of adult and infant anthropometric traits is being calibrated twice a week with the help of standard weights and measures.

Blood Pressure

Maternal blood pressure is being measured in the sitting position during each trimester after a rest of 5 minutes before taking the first reading, using an Omron blood pressure monitor (model: HBP-1300) validated for pregnant women[94] and cuff of appropriate size. Three readings will be taken with 2 minutes interval between the readings with maximum acceptable difference of 5mmHg between two readings. The equipment used is being calibrated thrice a week with the readings of mercury sphygmomanometer.

Biological sample collection and storage

A 15ml of blood sample is being collected during each trimester from the study participants after overnight fasting (10 hours). We are using EDTA coated, sodium fluoride coated and plain tubes for blood collection for the separation of EDTA-plasma, fluoride-plasma and serum samples, respectively. Serum will be used for determining

For peer review only - http://bmjopen.bmj.com/site/about/guidelines.xhtml

the levels of metabolites in addition to triglycerides, cholesterol, lipids, KFT, LFT and insulin whereas fluoride-plasma will be used for measuring glucose levels. EDTA tube is centrifuged at 3500 rpm for separating the WBCs (buffy coat), plasma and RBCs. After separating the plasma, EDTA tube containing WBCs will be stored for future extraction of DNA for genetic and epigenetic studies. Multiple aliquots of serum and plasma collected at each trimester are being stored in a -80°C freezer (Table-2).

We will collect cord blood and placenta at the time of delivery (Table-2) and processed with the help of the standard operating procedure used in the *"Avon Longitudinal Study of Parents and Children"* (ALSPAC), University of Bristol, UK. The cord blood will be collected in EDTA coated, heparin coated and plain tubes (10ml each) and these will be processed for the separation of plasma and serum. The remaining sample in EDTA tube with buffy coat will be stored in a freezer at -80°C. In order to stabilize the gene expression, RNA*later* will be added in Heparin tube. Placenta will be processed immediately after delivery and aliquots of tissue sample from maternal and foetal sides, cord tissue, and membrane will be stored in a freezers at -80°C (Table-2). All the stored aliquots will be appropriately bar-coded, stored independently in a separate storage box.

Insert Table-2

Laboratory tests

The fasting serum samples will be transferred to the Department of Anthropology, University of Delhi, on dry ice for the generation of data on biochemical assays on all the mothers during each trimester and also on their offspring using cord blood. The available auto-analyzer (XL-640, ERBA Mannheim, Czech Republic) in the Department of Anthropology, University of Delhi, India, will be used for biochemistry data generation (i.e. glucose, insulin, lipids, kidney function test (KFT) and liver function tests (LFT)) after ensuring daily calibration. Insulin levels will be estimated using ELISA kits. Further, a reagent lot calibration will also be performed whenever there is a change in lot number of the reagents to estimate the variation in assays between two reagent lots. For internal quality control, one blank per 100 samples and one control sample from *UK National External Quality Assessment Service* (UKNEQAS) of known concentrations will be run in every 200 samples. Moreover, one duplicate and one sample of known concentration from the previous batch will be run per 100 samples for assessing intra and inter assay variation, respectively, to help with analyses in confirming repeatability and reproducibility of biochemistry data. Finally, 5% of the samples will be run in a different laboratory for all the biochemical assays as a measure of external quality control. Moreover, information on participant's blood group, HbS and HIV infection,

For peer review only - http://bmjopen.bmj.com/site/about/guidelines.xhtml

which are the part of standard protocol of the hospital's antenatal care, will be taken from PBM Hospital.

Quantitative Metabolomics

The quantitative data on the level of metabolites (in mothers during each trimester and in offspring using cord blood) will be generated through a targeted approach for screening the predefined and known metabolites using a commercial assay i.e. AbsoluteIDQ p400 HR kit [Biocrates Life Sciences AG, Innsbruck, Austria]. It provides data on 400 endogenous metabolites from 11 metabolite classes (i.e. amino acids, biogenic amines, monosaccharides, acylcarnitines, diglycerides, triglycerides, lysophosphatidylcholines, phosphatidylcholines, sphingomyelins, ceramides, and cholesteryl esters). The choice of AbsoluteIDQ p400 HR kit based on proven utility of its previous version of kit (AbsoluteIDQ p180) in several metabolomics based epidemiological studies.[9,95,96,97,98] Our collaborator, the Phenome Centre Birmingham, University of Birmingham, UK, has experience of using this kit in an inter-laboratory trial. For generating data on targeted metabolites a Triple Quadrupole Mass Spectrometry based technology will be used which characterizes a metabolite by its molecular mass, its specific fractionation pattern (tandem mass spectrometry), and high sensitivity.[99] The complete analytical process will be performed using the MetIQ software package, which is an integral part of these kits. The quality control procedure used by Suhre et al.[100] will be followed for generation and cleaning of metabolomic data. One reference sample will be measured three times on each plate across the samples. The quality of metabolite data will be controlled by estimating their coefficient of variation and missing value rates.[15] Outlying samples and data points will be excluded and finally all missing values will be imputed.[101]

Body composition

The body composition of infants will be measured using a DXA machine (Osteo Pro Max, BM Technology, Korea) available in PBM Hospital, Bikaner. The machine will be calibrated using infant phantoms at regular intervals. We have developed a standard operating procedure with the help of published literature[102,103,104] for neonatal scans of femur, spine and whole body. Newborns will be measured within 48 hours of delivery after feeding in a calm or sleeping state. Infants will be swaddled in a thin cotton blanket in the required position and measurement will be taken without any metals, buttons or pins on the infant. The region of interest (ROI) for anterior-posterior spine will be five vertebral bodies of the lumber region; for femur scan this will include the femoral neck, trochanters, intertrochanteric, and total hip regions. For the whole body scan, the infant will be positioned at the centre of the table, aligned in a straight line from the nose, centre of the body, and down through the knees and toes. Six ROIs will be taken for the whole body scan (head, entire right and left arms, right and left leg regions and trunk).

For peer review only - http://bmjopen.bmj.com/site/about/guidelines.xhtml

Data capture, protection and management

The data is being recorded in paper based questionnaires that will be initially entered in Mircosoft Excel, and later transferred to a SQL based database to be developed in collaboration with the University of Bristol, UK. The master data will be stored at multiple locations (at least two) and managed in computers available in the Department of Anthropology, University of Delhi. One unique identification number (UID) is being assigned to each study participant (mothers) that will be common for her questionnaire-based information, biological measurements, blood and other biological samples. Similarly, newborns will also be assigned a UID which is linked to the UID of mothers. The computers will be password protected, and UID linked with personal identifiers necessary for contact and future follow up, will be encrypted and held separately in a different system and backed up regularly. All the biological samples will be collected in pre-assigned bar-coded tubes and vials and will be stored at -80°C in Bikaner, Rajasthan. A comprehensive data dictionary will be prepared for all the study variables and the entire data will be regularly checked for outliers, distribution of variables and missingness.

Data analyses

The evaluation of cross-sectional associations of maternal body weight with quantitative metabolite levels in each trimester, independently, will be done using regression-based modelling. Both level of metabolites and gestational weight in each trimester will act as exposure as well as outcome. Variables like socio-economic position, gestational age, parity, diet, smoking, and physical activity will be considered as confounders. Maternal characteristics will be considered as exposures (like trimester specific weight, metabolite levels) to evaluate their association with offspring related outcomes (foetal growth, birth weight, body compositions and metabolite levels). The potential confounders for maternal-offspring associations will be socio-economic position, gestational age, parity, diet, sex of the offspring, smoking and physical activity.

Mixed effects regression models for repeated measures will be applied for examining longitudinal associations of weight gain from first to third trimester[42] with maternal levels of metabolites during pregnancy and foetal growth.[105] We will also analyse the associations of level of metabolites from first to third trimester with trajectories of gestational weight gain. Mediation analyses will be conducted for evaluating the role of maternal metabolites as intermediates between gestational weight gain and offspring's body composition.[106] False discovery rate based multiple testing correction for number of metabolites will be applied.[107] Mediation analysis will be conducted to examine whether the effect of GWG on foetal growth is mediated through alteration in levels of circulating metabolites.

For peer review only - http://bmjopen.bmj.com/site/about/guidelines.xhtml

Public health implications

In India, higher prevalence of both malnutrition and over-nutrition,[108,109] increased rates of gestational diabetes[110] and pre-eclampsia[111] are common challenges related to maternal-foetal health. Moreover, India has high rates of birth defects, i.e. over 60 per 1000 live births[112], higher number of infants born small for gestational age,[75] still births,[113] and more than 45% of children under 5 years of age are stunted[114] which need to be addressed through translational research. The metabolomic approach will enable the identification of biochemical markers predictive for undesirable birth outcomes.

The role of the intrauterine environment, through foetal overnutrition, has been proposed in considering determination of higher birth weight, childhood obesity, impaired glucose tolerance, and dyslipidemia[115,116,117]. The knowledge of trimester-specific quantitative levels of metabolites will uncover mechanistic pathways relevant for optimum foetal growth and healthy birth outcomes. Understanding of changes in gestational metabolism will provide many opportunities for detecting potentially relevant metabolites for planning effective interventions during pregnancy.

Ethics and dissemination

The study has been approved by the ethics committees of the Department of Anthropology, University of Delhi and Sardar Patel Medical College, Bikaner, Rajasthan. We are taking written informed consent after discussing the various aspects of the study with the participants in the local language. The findings of the study will be disseminated through peer reviewed national and international journals and through conferences and seminars.

Acknowledgements

We are thankful to the study participants, project’s field investigators, medical staff and administration of PBM hospital, Bikaner, Rajasthan for facilitating the study.

Authors’ Contributions

VG conceived the study concept with the help of GDS. VG developed the funding proposal, applied for funding and initiated the writing of the protocol paper. VG, GKW and MPS developed the study tools and procedures. VG, RS, RK, MPS and GKW coordinated the implementation and running of the study. GDS has advised in developing the study protocols and quality control and assurances. WD has provided expert opinion on metabolomic data generation and quality control. AP has provided expert opinion on ultrasound measures. GDS, CR, US, WD and VG have contributed to

For peer review only - http://bmjopen.bmj.com/site/about/guidelines.xhtml

the statistical analysis plan and data interpretation. VG, RS, GKW, RK, TA and HV contributed to data collection and field management. All authors contributed in the revision and approval of the final version of the manuscript.

	Mother			
	1st Trimester	2nd Trimester	3rd Trimester	Offspring
**Timing of data collection**	10th to 13th weeks	22nd to 24th weeks	32nd to 34th weeks	Birth
**Gynaecological and Obstetric**	✔	✔	✔	-

Funder's statement: This study has been supported by Wellcome/DBT India Alliance, Hyderabad, India, "Intermediate" fellowship award to Vipin Gupta [grant reference: IA/CPHI/16/1/502623]. ATP is supported by the Oxford Partnership Comprehensive Biomedical Research Centre with funding from the NIHR Biomedical Research Centres funding scheme.

Competing interest statement: None

For peer review only

Table-1:Measures, tools and time points of data collection

For peer review only - http://bmjopen.bmj.com/site/about/guidelines.xhtml

**Socio-economic position**	✔	✔	✔	-
**Anthropological (caste and religion)**	✔	✔	✔	-
**Education**	✔	✔	✔	-
**Marital status**	✔	✔	✔	-
**Smoking**	✔	✔	✔	-
**Alcohol drinking**	✔	✔	✔	-
**Medication**	✔	✔	✔	-
**Disease history**	✔	✔	✔	-
**Maternal Environment**	✔	✔	✔	-
**Physical activity (IPAQ)**	✔	✔	✔	-
**Time-Activity pattern**	✔	✔	✔	-
**Food frequency**	✔	✔	✔	-
**Dietary recall (3 days)**	✔	✔	✔	-
**PHQ-12**	✔	✔	✔	-
**Wellbeing (WHO-5)**	✔	✔	✔	-
**Depression (EPNDS)**	✔	✔	✔	-
**Social factors**	✔	✔	✔	-
**Capability wellbeing (ICECAP-A)**	✔	✔	✔	-
**Anthropometry**				
Height/Body length	✔	✔	✔	✔
Sitting height	✔	✔	✔	-
Weight	✔	✔	✔	✔
Circumferences	✔	✔	✔	✔
Skin-folds	✔	✔	✔	✔
**Blood Pressure**	✔	✔	✔	-
**Laboratory tests:** Glucose, Insulin, Hb, Lipids, LFT and KFT, and blood group	✔	✔	✔	✔
**Metabolomic data**	✔	✔	✔	✔
**Ultrasound measures:** CRL, BPD, OFD, HC, FL, AC and APAD	✔	✔	✔	-
**Placenta and cord measures and images**	-	-	-	✔
**Body Composition (DXA Scan)**	-	-	-	✔

IPAQ: International Physical Activity Questionnaire; PHQ: Psychological health Questionnaire; WHO: World Health Organization; ICECAP-A: ICEpop CAPability measure for Adults; EPNDS: Edinburg Post Natal Depression Scale; LFT: liver function tests; KFT: kidney function tests; CRL: Crown-rump length; BPD: Biparietal diameter; OFD: Occipito-frontal diameter; HC: Head Circumference; FL: Femur length; APAD: Anterior-posterior abdominal diameter

Table-2: Type and number of aliquots of biological samples

	**Pregnancy**	

For peer review only - http://bmjopen.bmj.com/site/about/guidelines.xhtml

	1st Trimester	2nd Trimester	3rd Trimester	Offspring
**Blood Samples**				
Serum	**6 aliquots**	**6 aliquots**	**6 aliquots**	**-**
EDTA Plasma	**2 aliquots**	**2 aliquots**	**2 aliquots**	**-**
Fluoride Plasma	**2 aliquots**	**2 aliquots**	**2 aliquots**	**-**
Buffy coat	**1 aliquot**	**1 aliquot**	**1 aliquot**	**-**
**Cord Blood Samples**				
Serum	**-**	**-**	**-**	**4 aliquots**
EDTA Plasma	**-**	**-**	**-**	**3 aliquots**
Heparin Plasma	**-**	**-**	**-**	**3 aliquots**
Buffy coat with RNA*later*	**-**	**-**	**-**	**1 aliquot**
Buffy coat	**-**	**-**	**-**	**1 aliquot**
**Placenta Samples**				
Maternal side tissue	**-**	**-**	**-**	**8 aliquots**
Maternal side tissue with RNA*later*	**-**	**-**	**-**	**4 aliquots**
Foetal side tissue	**-**	**-**	**-**	**1 aliquot**
Foetal side tissue with RNA*later*	**-**	**-**	**-**	**1 aliquot**
Membrane tissue	**-**	**-**	**-**	**1 aliquot**
Cord tissue	**-**	**-**	**-**	**1 aliquot**
Cord tissue with RNA*later*	**-**	**-**	**-**	**1 aliquot**

For peer review only

For peer review only - http://bmjopen.bmj.com/site/about/guidelines.xhtml

References

1. Picciano MF. Pregnancy and lactation: physiological adjustments, nutritional requirements and the role of dietary supplements. *J Nutr* 2003;133:1997S-2002S.
2. Lowe WL, Karban J. Genetics, genomics and metabolomics: new insights into maternal metabolism during pregnancy. *Diabet Med* 2014;31:254-62.
3. Barker DJP. In utero programming of chronic disease. *Clin Sci (Lond)* 1998;95:115-28.
4. Gluckman PD, Hanson MA. Living with the past: evolution, development, and patterns of disease. *Science* 2004;305:1733–6.
5. Carreras-Badosa G, Prats-Puig A, Puig T, et al. Circulating Fatty Acid Synthase in pregnant women: Relationship to blood pressure, maternal metabolism and newborn parameters. *Sci Rep* 2016;6:24167.
6. Hales CN, Barker DJ. The thrifty phenotype hypothesis. *Br Med Bull* 2001;60:5-20.
7. Van Assche FA, Holemans K, Aerts L. Long-term consequences for offspring of diabetes during pregnancy. *Br Med Bull* 2001;60:173-82.
8. Lawlor DA, Lichtenstein P, Fraser A, et al. Does maternal weight gain in pregnancy have long-term effects on offspring adiposity? A sibling study in a prospective cohort of 146,894 men from 136,050 families. *Am J Clin Nutr* 2011;94:142-8.
9. Gieger C, Geistlinger L, Altmaier E, et al. Genetics meets metabolomics: a genomewide association study of metabolite profiles in human serum. *PLoS Genet* 2008;4:e1000282.
10. Suhre K, Gieger C. Genetic variation in metabolic phenotypes: study designs and applications. *Nat Rev Genet* 2012;13:759-69.
11. Wishart DS, Jewison T, Guo AC, et al. HMDB 3.0--The Human Metabolome Database in 2013. *Nucleic Acids Res* 2013;41:D801-7.
12. Nicholson G, Rantalainen M, Li JV, et al. A genome-wide metabolic QTL analysis in Europeans implicates two loci shaped by recent positive selection. *PLoS Genet* 2011;7:e1002270.
13. Fiehn O. Metabolomics--the link between genotypes and phenotypes. *Plant Mol Biol* 2002;48:155-71.
14. Psychogios N, Hau DD, Peng J, et al. The human serum metabolome. *PLoS One* 2011;6:e16957.
15. Lindsay KL, Hellmuth C, Uhl O, et al. Longitudinal Metabolomic Profiling of Amino Acids and Lipids across Healthy Pregnancy. *PLoS One* 2015;10:e0145794.

For peer review only

For peer review only - http://bmjopen.bmj.com/site/about/guidelines.xhtml

16. Maitre L, Villanueva CM, Lewis MR, et al. Maternal urinary metabolic signatures of fetal growth and associated clinical and environmental factors in the INMA study. *BMC Med* 2016;14:177.
17. Gong S, Sovio U, Aye ILMH, et al. Placental polyamine metabolism differs by fetal sex, fetal growth restriction and preeclampsia. *JCI Insight* 2018; 3(13):e120723.
18. Ruchat SM, Hivert MF, Bouchard L. Epigenetic programming of obesity and diabetes by in utero exposure to gestational diabetes mellitus. *Nutr Rev* 2013;71:S88-94.
19. Gao J, Xu B, Zhang X, et al. Association between serum bile acid profiles and gestational diabetes mellitus: A targeted metabolomics study. *Clin Chim Acta* 2016;459:63-72.
20. Lu L, Koulman A, Petry CJ, et al. An Unbiased Lipidomics Approach Identifies Early Second Trimester Lipids Predictive of Maternal Glycemic Traits and Gestational Diabetes Mellitus. *Diabetes Care* 2016;39:2232-2239.
21. Gelaye B, Sumner SJ, McRitchie S, et al. Maternal Early Pregnancy Serum Metabolomics Profile and Abnormal Vaginal Bleeding as Predictors of Placental Abruption: A Prospective Study. *PLoS One* 2016;11:e0156755.
22. Li J, Lu YP, Reichetzeder C, et al. Maternal PCaaC38:6 is Associated With Preterm Birth - a Risk Factor for Early and Late Adverse Outcome of the Offspring. *Kidney Blood Press Res* 2016;41:250-7.
23. Powell KL, Carrozzi A, Stephens AS, et al. Utility of metabolic profiling of serum in the diagnosis of pregnancy complications. *Placenta* 2018;66:65-73.
24. Dessì A, Marincola FC, Pattumelli MG, et al. Investigation of the ^{1}H-NMR based urine metabolomic profiles of IUGR, LGA and AGA newborns on the first day of life. *J Matern Fetal Neonatal Med* 2014;27:13-9.
25. Chorell E, Hall UA, Gustavsson C, Berntorp K, Puhkala J, Luoto R, Olsson T, Holmäng A. Pregnancy to postpartum transition of serum metabolites in women with gestational diabetes. Metabolism. 2017 Jul;72:27-36.
26. Lain KY, Catalano PM. Metabolic changes in pregnancy. *Clin Obstet Gynecol* 2007;50:938–48.
27. Diareme M, Karkalousos P, Theodoropoulos G, et al. Lipid profile of healthy women during normal pregnancy. *Journal of Medical Biochemistry* 2009;28:152–60.
28. IOM (Institute of Medicine)/NRC (National Research Council). Weight gain during pregnancy: reexamining the guidelines. In: Rasmussen KM, Yaktine AL, eds. *Food and Nutrition Board and Board on Children, Youth, and Families*. National Academies Press (US), 2009.
29. Fraser A, Tilling K, Macdonald-Wallis C, et al. Associations of gestational weight gain with maternal body mass index, waist circumference, and blood pressure

For peer review only - http://bmjopen.bmj.com/site/about/guidelines.xhtml

measured 16y after pregnancy: the Avon Longitudinal Study of Parents and Children (ALSPAC). *Am J Clin Nutr* 2011;93:1285-92.

30. Poston L. Gestational weight gain: influences on the long-term health of the child. *Curr Opin Clin Nutr Metab Care* 2012;15:252-7.
31. Drehmer M, Duncan BB, Kac G, et al. Association of second and third trimester weight gain in pregnancy with maternal and fetal outcomes. *PLoS One* 2013;8:e54704.
32. Goldstein RF, Abell SK, Ranasinha S, et al. Association of Gestational Weight Gain With Maternal and Infant Outcomes: A Systematic Review and Meta-analysis. *JAMA* 2017;317:2207-2225.
33. Mannan M, Doi SA, Mamun AA. Association between weight gain during pregnancy and postpartum weight retention and obesity: a bias-adjusted meta-analysis. *Nutr Rev* 2013;71:343–52.
34. Oken E, Kleinman KP, Belfort MB, et al. Associations of gestational weight gain with short- and longer-term maternal and child health outcomes. *Am J Epidemiol* 2009;170:173-80.
35. Hellmuth C, Lindsay KL, Uhl O, et al. Association of maternal prepregnancy BMI with metabolomic profile across gestation. *Int J Obes (Lond)* 2017;41:159-169.
36. Lawlor DA. The Society for Social Medicine John Pemberton Lecture 2011. Developmental overnutritiondan old hypothesis with new importance? *Int J Epidemiol* 2013;42:7e29.
37. Fraser A, Tilling K, Macdonald-Wallis C, et al. Association of maternal weight gain in pregnancy with offspring obesity and metabolic and vascular traits in childhood. *Circulation* 2010;121:2557-64.
38. Karachaliou M, Georgiou V, Roumeliotaki T, et al. Association of trimester-specific gestational weight gain with fetal growth, offspring obesity, and cardiometabolic traits in early childhood. *Am J Obstet Gynecol* 2015;212:502.e1-14.
39. Davenport MH, Ruchat SM, Giroux I, et al. Timing of excessive pregnancy-related weight gain and offspring adiposity at birth. *Obstet Gynecol* 2013;122:255e61.
40. Gaillard R, Steegers EA, Franco OH, et al. Maternal weight gain in different periods of pregnancy and childhood cardio-metabolic outcomes. The Generation R Study. *Int J Obes (Lond)* 2015;39:677-85.
41. Ruchat SM, Allard C, Doyon M, et al. Timing of Excessive Weight Gain During Pregnancy Modulates Newborn Anthropometry. *J Obstet Gynaecol Can* 2016;38:108-17.
42. Cheikh Ismail Leila, Bishop D C, Pang R, et al. Gestational weight gain standards based on women enrolled in the Fetal Growth Longitudinal Study of the

For peer review only - http://bmjopen.bmj.com/site/about/guidelines.xhtml

INTERGROWTH-21st Project: a prospective longitudinal cohort study. *BMJ* 2016; 352 :i555

43. Pitkin RM. Nutritional support in obstetrics and gynecology. *Clin Obstet Gynecol* 1976;19:489-513.
44. Nelson SM, Matthews P, Poston L. Maternal metabolism and obesity: modifiable determinants of pregnancy outcome. *Hum Reprod Update* 2010;16:255-75.
45. Radhakrishnan U, Kolar G, Nirmalan PK. Cross-sectional study of gestational weight gain and perinatal outcomes in pregnant women at a tertiary care center in southern India. *J Obstet Gynaecol Res.* 2014;40(1):25-31.
46. Mahalakshmi MM, Bhavadharini B, Kumar M, et al. Clinical profile, outcomes, and progression to type 2 diabetes among Indian women with gestational diabetes mellitus seen at a diabetes center in south India. *Indian J Endocrinol Metab.* 2014;18(3):400-6.
47. Mukhopadhyay A, Dwarkanath P, Bhanji S, et al. Maternal intake of milk and milk proteins is positively associated with birth weight: A prospective observational cohort study. *Clin Nutr ESPEN.* 2018;25:103-109.
48. Hickey CA, Cliver SP, Goldenberg RL, et al. Relationship of psychosocial status to low prenatal weight gain among nonobese black and white women delivering at term. *Obstet Gynecol* 1995;86:177-183.
49. Olson CM, Strawderman MS. Modifiable behavioral factors in a biopsychosocial model predict inadequate and excessive gestational weight gain. *J Am Diet Assoc* 2003;103:48-54.
50. Brawarsky P, Stotland NE, Jackson RA, et al. Pre-pregnancy and pregnancy-related factors and the risk of excessive or inadequate gestational weight gain. *Int J Gynaecol Obstet* 2005;91:125-131.
51. Perng W, Gillman MW, Fleisch AF, et al. Metabolomic profiles and childhood obesity. *Obesity (Silver Spring)* 2014;22:2570-8.
52. Santos Ferreira DL, Williams DM, Kangas AJ, et al. Association of pre-pregnancy body mass index with offspring metabolic profile: Analyses of 3 European prospective birth cohorts. *PLoS Med* 2017;14:e1002376.
53. Tyrrell J, Richmond RC, Palmer TM. Genetic Evidence for Causal Relationships between Maternal Obesity-Related Traits and Birth Weight. *JAMA* 2016;315:1129-40.
54. Ngo TTM, Moufarrej MN, Rasmussen MH et al. Noninvasive blood tests for fetal development predict gestational age and preterm delivery. Science 2018; 360 (6393): 1133-1136.
55. Patel N, Hellmuth C, Uhl O. Cord Metabolic Profiles in Obese Pregnant Women: Insights Into Offspring Growth and Body Composition. *J Clin Endocrinol Metab* 2018;103:346-355.

For peer review only - http://bmjopen.bmj.com/site/about/guidelines.xhtml

56. Scholtens DM, Bain JR, Reisetter AC, et al. Metabolic Networks and Metabolites Underlie Associations Between Maternal Glucose During Pregnancy and Newborn Size at Birth. *Diabetes* 2016;65:2039-50.
57. Freinkel N. Banting Lecture 1980. Of pregnancy and progeny. *Diabetes*1980;29:1023–1035.
58. Maitre L, Fthenou E, Athersuch T, et al. Urinary metabolic profiles in early pregnancy are associated with preterm birth and fetal growth restriction in the Rhea mother-child cohort study. *BMC Med* 2014;12:110.
59. Census of India, 2011, Rajasthan, http://www.rajcensus.gov.in/).
60. Annual Health Survey (2011-12). Vital Statistics Division Office of the Registrar General & Census Commissioner, India New Delhi.
61. Ciborowski M, Zbucka-Kretowska M, Bomba-Opon D, et al. Potential first trimester metabolomic biomarkers of abnormal birth weight in healthy pregnancies. *Prenat Diagn* 2014;34:870-7.
62. Kenny LC, Broadhurst DI, Dunn W, et al. Robust Early Pregnancy Prediction of Later Preeclampsia Using Metabolomic Biomarkers. *Hypertension* 2010;56:741−49.
63. Zheng X, Su M, Pei L, et al. Metabolic signature of pregnant women with neural tube defects in offspring. *J. Proteome Res* 2011;10:4845−54.
64. Bahado-Singh RO, Akolekar R, Mandal R, et al. First-trimester metabolomic detection of late-onset preeclampsia. *Am J Obstet Gynecol* 2013;208:58.e1−7.
65. Paine MA, Scioscia M, Williams PJ, et al. Urinary inositol phosphoglycan P-type as a marker for prediction of preeclampsia and novel implications for the pathophysiology of this disorder. *Hypertens Pregnancy* 2010;29:375–84.
66. Dessi A, Atzori L, Noto A, et al. Metabolomics in newborns with intrauterine growth retardation (IUGR): urine reveals markers of metabolic syndrome. *J Matern Fetal Neonatal Med* 2011;2:35-9.
67. Ivorra C, García-Vicent C, Chaves FJ, et al. Metabolomic profiling in blood from umbilical cords of low birth weight newborns. *J Transl Med* 2012;10:142.
68. Walsh JM, Wallace M, Brennan L, et al. Early pregnancy maternal urinary metabolomic profile and later insulin resistance and fetal adiposity. *J Matern Fetal Neonatal Med* 2015;28:1697-700.
69. Tzoulaki I, Ebbels TM, Valdes A, et al. Design and analysis of metabolomics studies in epidemiologic research: a primer on – omic technologies. *Am J Epidemiol* 2014;180:129-39.
70. Tuulia Tynkkynen T, Wang Q, Ekholm J, Anufrieva O et al. Proof of concept for quantitative urine NMR metabolomics pipeline for large-scale epidemiology and genetics. BioRxiv. doi: https://doi.org/10.1101/288993

For peer review only - http://bmjopen.bmj.com/site/about/guidelines.xhtml

71. Pinto J, Barros AS, Domingues MR, et al. Following healthy pregnancy by NMR metabolomics of plasma and correlation to urine. *J Proteome Res* 2015;14:1263-74.
72. Odegård RA, Vatten LJ, Nilsen ST, et al. Preeclampsia and fetal growth. *Obstet Gynecol* 2000;96:950-5.
73. Liu F, Zhao C, Liu L, et al. Peptidome profiling of umbilical cord plasma associated with gestational diabetes-induced fetal macrosomia. *J Proteomics* 2016;139:38-44.
74. Lampl M, Kusanovic JP, Erez O, et al. Early rapid growth, early birth: accelerated fetal growth and spontaneous late preterm birth. *Am J Hum Biol* 2009;21:141-50.
75. Lee AC, Katz J, Blencowe H, et al. National and regional estimates of term and preterm babies born small for gestational age in 138 low-income and middle-income countries in 2010. *Lancet Glob Health* 2013;1:e26-36.
76. Papageorghiou AT, Kennedy SH, Salomon LJ, et al. International Fetal and Newborn Growth Consortium for the 21st Century (INTERGROWTH-21st). International standards for early fetal size and pregnancy dating based on ultrasound measurement of crown-rump length in the first trimester of pregnancy. *Ultrsound Obstet Gyneco* 2014;44:641-8.
77. Stirnemann J, Villar J, Salomon LJ, et al. International estimated fetal weight standards of the INTERGROWTH-21st Project. *Ultrasound Obstet Gynecol* 2017;49:478-486.
78. National Institute of Health and Care Excellence (NICE), National Collaborating Centre for Women's and Children's Health. NICE guideline: antenatal care. London: Royal College of Obstetricians and Gynaecologists Press, 2008.
79. Ebrahim S, Kinra S, Bowen L, et al. The effect of rural-to-urban migration on obesity and diabetes in India: a cross-sectional study. *PLoS Med* 2010;7:e1000268.
80. Hagströmer M, Oja P, Sjöström M. The International Physical Activity Questionnaire (IPAQ): A study of concurrent and construct validity. *Public Health Nutr* 2006;9:755-62.
81. Bertolotto A, Volpe L, Calianno A, et al. Physical activity and dietary habits during pregnancy: effects on glucose tolerance. *J Matern Fetal Neonatal Med* 2010;23:1310-4.
82. Harizopoulou VC, Kritikos A, Papanikolaou Z, et al. Maternal physical activity before and during early pregnancy as a risk factor for gestational diabetes mellitus. *Acta Diabetol* 2010;47:83-9.
83. International Institute for Population Sciences (IIPS) and ICF. 2017. National Family Health Survey (NFHS-4), 2015-16: India. Mumbai: IIPS. http://www.rchiips.org/nfhs/nfhs4.shtml.

For peer review only - http://bmjopen.bmj.com/site/about/guidelines.xhtml

84. Poongothai S, Pradeepa R, Ganesan A, et al. Reliability and validity of a modified PHQ-9 item inventory (PHQ-12) as a screening instrument for assessing depression in Asian Indians (CURES-65). *J Assoc Physicians India* 2009;57:147-52.
85. Shivalli S, Gururaj N. Postnatal depression among rural women in South India: do socio-demographic, obstetric and pregnancy outcome have a role to play? *PLoS One* 2015;10:e0122079.
86. Topp CW, Østergaard SD, Søndergaard S, et al. The WHO-5 Well-Being Index: a systematic review of the literature. *Psychother Psychosom* 2015;84:167-76.
87. Al-Janabi H, Flynn TN, Coast J. Development of a self-report measure of capability wellbeing for adults: the ICECAP-A. *Qual Life Res* 2012;21:167-76.
88. Ilias Goranitis, Joanna Coast, Hareth Al-Janabi, et al. The validity and responsiveness of the ICECAP-A capability-well-being measure in women with irritative lower urinary tract symptoms. *Qual Life Res* 2016;25:2063–2075.
89. Keeley T, Al-Janabi H, Nicholls E, et al. A longitudinal assessment of the responsiveness of the ICECAP-A in a randomised controlled trial of a knee pain intervention. *Qual Life Res.* 2015;24:2319–2331.
90. Villar J, Altman DG, Purwar M, et al. The objectives, design and implementation of the INTERGROWTH-21st Project. *BJOG* 2013; 120:9-26.
91. Eskenazi B, Bradman A, Finkton D, et al. A rapid questionnaire assessment of environmental exposures to pregnant women in the INTERGROWTH 21st Project. *BJOG* 2013;120:129-38,v.
92. The International Society for the Advancement of Kinanthropometry. *International Standards for Anthropometric Assessment*. Australia: ISAK, 2001.
93. Cheikh Ismail L, Knight HE, Bhutta Z, et al. International Fetal and Newborn Growth Consortium for the 21st Century. Anthropometric protocols for the construction of new international fetal and newborn growth standards: the INTERGROWTH-21st Project. *BJOG* 2013;120:42-7.
94. Abbud L, Nzelu D, Salaria M, et al. Validation of the Omron HBP-1300 in pregnancy for medium-arm and large-arm circumferences according to the British Hypertension Society protocol. *Blood Press Monit.* 2018;23(5):277-280.
95. Illig T, Gieger C, Zhai G, et al. A genome-wide perspective of genetic variation in human metabolism. *Nat Genet* 2010;42:137-41.
96. Altmaier E, Emeny RT, Krumsiek J, et al. Metabolomic profiles in individuals with negative affectivity and social inhibition: a population-based study of Type D personality. *Psychoneuroendocrinology* 2013;38:1299-309.
97. Breier M, Wahl S, Prehn C. Targeted metabolomics identifies reliable and stable metabolites in human serum and plasma samples. *PLoS One* 2014;9:e89728.

For peer review only - http://bmjopen.bmj.com/site/about/guidelines.xhtml

98. Petersen AK, Zeilinger S, Kastenmüller G, et al. Epigenetics meets metabolomics: an epigenome-wide association study with blood serum metabolic traits. *Hum Mol Genet* 2014;23:534-45.
99. Johnson CH, Gonzalez FJ. Challenges and opportunities of metabolomics. *J Cell Physiol* 2012;227:2975-81.
100. Suhre K, Shin SY, Petersen AK, et al. Human metabolic individuality in biomedical and pharmaceutical research. *Nature* 2011;477:54-60.
101. Liao SG, Lin Y, Kang DD, et al. Missing value imputation in high-dimensional phenomic data: imputable or not, and how? *BMC Bioinformatics* 2014;15:346.
102. Eichenberger Gilmore JM, Pauley CA, Burns TL, et al. A hip analysis protocol for pediatric bone desnsitometry: the Iowa Bne Development Study. *J Clin Densitom* 2010;13:361-9.
103. International Atomic Energy Agency, 2013. Body composition assessment from birth to 2 years of age. Vienna, IAEA human health series ISSN 2075-3772 no-22.
104. Shepherd JA, Sommer MJ, Fan B, et al. Advanced Analysis Techniques Improve Infant Bone and Body Composition Measures by Dual-Energy X-Ray Absorptiometry. *J Pediatr* 2017;181:248-253.e3.
105. Nakai M, Ke W. Statistical Models for Longitudinal Data Analysis. *Applied Mathematical Sciences* 2009;3:1979–89.
106. Baron RM, Kenny DA. The moderator-mediator variable distinction in social psychological research: conceptual, strategic, and statistical considerations. *J Pers Soc Psychol* 1986;51:1173–82.
107. Storey JD. The positive false discovery rate: A Bayesian interpretation and the q-value. *The Annals of Statistics* 2003;31:2013–35.
108. Razak F, Corsi DJ, Subramanian SV. Change in the body mass index distribution for women: analysis of surveys from 37 low- and middle-income countries. *PLoS Med* 2013;10:e1001367.
109. Jones AD, Hayter AK, Baker CP. The co-occurrence of anemia and cardiometabolic disease risk demonstrates sex-specific socio demographic patterning in an urbanizing rural region of southern India. *Eur J Clin Nutr* 2016;70:364-72.
110. Mithal A, Bansal B, Kalra S. Gestational diabetes in India: Science and society. Indian *J Endocrinol Metab* 2015;19:701-4.
111. Agrawal S, Walia GK. Prevalence and Risk Factors for Symptoms Suggestive of PreEclampsia in Indian Women. *Journal of Womens Health Issues Care* 2014;3:6.
112. Sharma R. Birth defects in India: Hidden truth, need for urgent attention. *Indian J Hum Genet* 2013;19:125-9.

For peer review only - http://bmjopen.bmj.com/site/about/guidelines.xhtml

113. Bhati DK. Stillbirths: A high magnitude public health issue in India. *South East Asia Journal Of Public Health* 2013;3:3-9.
114. Subramanyam MA, Kawachi I, Berkman LF, et al. Is economic growth associated with reduction in child undernutrition in India? *PLoS Med* 2011;8:e1000424.
115. Metzger BE. Long-term outcomes in mothers diagnosed with gestational diabetes mellitus and their offspring. *Clin Obstet Gynecol* 2007;50:972–979.
116. Dabelea D, Crume T. Maternal environment and the transgenerational cycle of obesity and diabetes. *Diabetes* 2011;60:1849–1855.
117. Catalano PM, Hauguel-De Mouzon S. Is it time to revisit the Pedersen hypothesis in the face of the obesity epidemic? *Am J Obstet Gynecol* 2011;204:479–487.

Figure legends:

Figure-1: Diagrammatic representation of hypothesis: *Quantitative changes in maternal circulating metabolites associated with gestational weight gain, influences foetal growth, offspring's levels of metabolites, body composition and anthropometric traits at birth.*

Figure-2: Recruitment and follow-up strategy

For peer review only

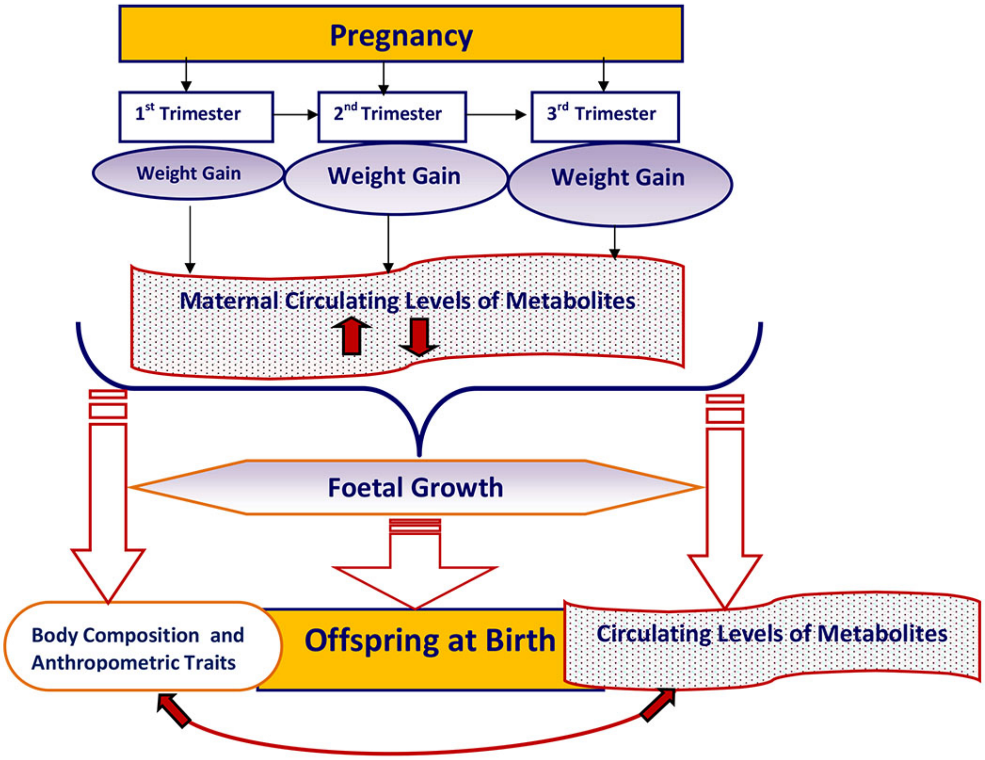

Figure-1: Diagrammatic representation of hypothesis: Quantitative changes in maternal circulating metabolites associated with gestational weight gain, influences foetal growth, offspring's levels of metabolites, body composition and anthropometric traits at birth.

90x90mm (300 x 300 DPI)

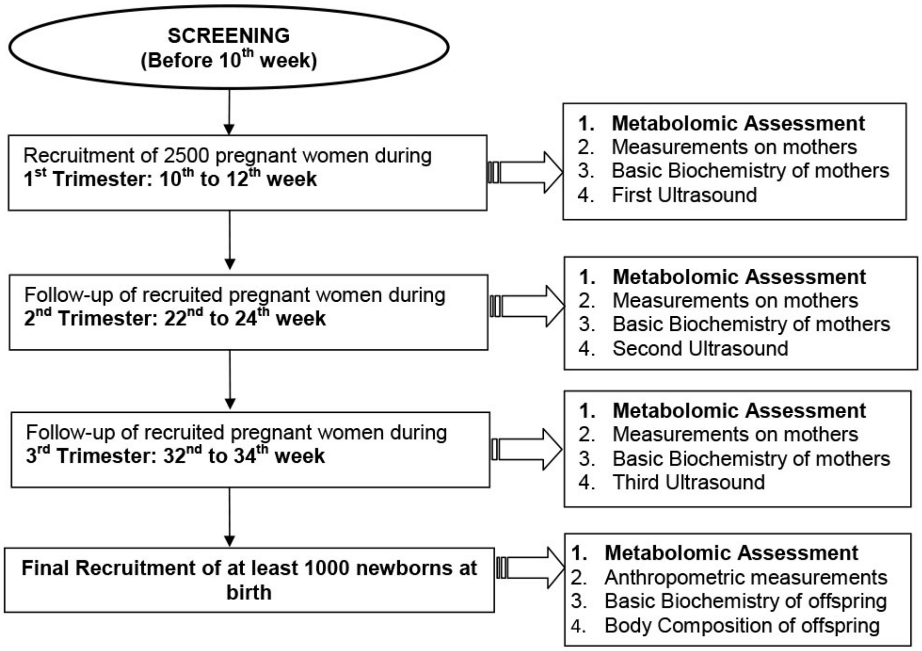

Figure-2: Recruitment and follow-up strategy

90x90mm (300 x 300 DPI)
